# Learning a Health Knowledge Graph from Electronic Medical Records

**DOI:** 10.1038/s41598-017-05778-z

**Published:** 2017-07-20

**Authors:** Maya Rotmensch, Yoni Halpern, Abdulhakim Tlimat, Steven Horng, David Sontag

**Affiliations:** 1 Center for Data Science, New York University, New York, NY USA; 2 Department of Computer Science, New York University, New York, NY USA; 3 Department of Emergency Medicine, Beth Israel Deaconess Medical Center, Boston, MA USA; 4 Division of Clinical Informatics, Beth Israel Deaconess Medical Center, Boston, MA USA; 5 Department of Electrical Engineering and Computer Science, Computer Science and Artificial Intelligence Laboratory, Massachusetts Institute of Technology, Cambridge, MA USA; 6 Institute for Medical Engineering & Science Massachusetts Institute of Technology, Cambridge, MA USA

## Abstract

Demand for clinical decision support systems in medicine and self-diagnostic symptom checkers has substantially increased in recent years. Existing platforms rely on knowledge bases manually compiled through a labor-intensive process or automatically derived using simple pairwise statistics. This study explored an automated process to learn high quality knowledge bases linking diseases and symptoms directly from electronic medical records. Medical concepts were extracted from 273,174 de-identified patient records and maximum likelihood estimation of three probabilistic models was used to automatically construct knowledge graphs: logistic regression, naive Bayes classifier and a Bayesian network using noisy OR gates. A graph of disease-symptom relationships was elicited from the learned parameters and the constructed knowledge graphs were evaluated and validated, with permission, against Google’s manually-constructed knowledge graph and against expert physician opinions. Our study shows that direct and automated construction of high quality health knowledge graphs from medical records using rudimentary concept extraction is feasible. The noisy OR model produces a high quality knowledge graph reaching precision of 0.85 for a recall of 0.6 in the clinical evaluation. Noisy OR significantly outperforms all tested models across evaluation frameworks (p < 0.01).

## Introduction

Automated tools to support medical diagnostic reasoning are used by patients seeking information about their symptoms^1–4^, as well as by clinicians when faced with a difficult case or to avoid prematurely focusing on a small number of potential diagnoses^5^. Considerable effort has been put into building diagnostic reasoning systems and encoding relevant information to drive their inference capabilities^6–11^. These models showed significant success in improving didactic practices^12, 13^, assisting with diagnosis^6, 7, 9, 11, 14^, and at times even outperforming experienced doctors^15^.

Historically, the models used by diagnostic reasoning systems were specified manually, requiring tremendous amounts of expert time and effort. For example, it was estimated that about fifteen person-years were spent building the Internist-1/QMR knowledge base for internal medicine^10^. However, the manual specification made these models extremely brittle and difficult to adapt to new diseases or clinical settings. Automatic compilation of a graph relating diseases to the symptoms that they cause has the potential to significantly speed up the development of such diagnosis tools. Moreover, such graphs would provide value in and of themselves. For example, given that general-purpose web-search engines are among the most commonly consulted sources for medical information^4, 16^, health panels such as those provided by Google using their health knowledge graph have a tremendous potential for impact^17–19^.

Previous work considered the use of natural language processing to find relationships between diseases and symptoms from unstructured or semi-structured data. For example, IBM’s WatsonPaths and the symptom checker Isabel made use of medical textbooks, journals, and trusted web content^8, 9^. However, another potential source of data, currently underutilized, is the electronic medical record (EMR), which has become increasingly prevalent in the United States and worldwide^20^.

EMR data is difficult to interpret for four main reasons: First, the text of physician and nursing notes is less formal than that of traditional textbooks, making it difficult to consistently identify disease and symptom mentions. Second, textbooks and journals often present simplified cases that relay only the most typical symptoms, to promote learning. EMR data presents real patients with all of the comorbidities, confounding factors, and nuances that make them individuals. Third, unlike textbooks that state the relationships between diseases and symptoms in a declarative manner, the associations between diseases and symptoms in the EMR are statistical, making it easy to confuse correlation with causation. Finally, the manner in which observations are recorded in the EMR is filtered through the decision-making process of the treating physician. Information deemed irrelevant may be omitted or not pursued, leading to information missing not at random^21^.

Despite EMR data being more difficult to work with for the reasons described above, it has the advantage of being closer to the actual practice of medicine than the idealized and curated information presented in textbooks and journals. For example, learning from EMRs provides the opportunity to discover new relationships that were previously unrecognized. Additionally, we can learn specialized graphs with different granularity for different specialties or settings by simply learning models from the records of patients from that setting. Finally, learning a graph of candidate causal relations involving diseases and symptoms from EMRs is the first step toward learning models that perform diagnostic inference directly from the real data that is continuously being generated from the healthcare system.

## Contributions

We present a methodology for automatically deriving a graph relating diseases to the symptoms that they might cause from EMR data. We evaluate the methodology by learning a graph tailored to an acute care setting from emergency department records of over 270,000 patient visits. By evaluating the learned graph against physicians’ expert opinion and comparing our performance against the performance of the Google health knowledge graph, we demonstrate the viability of producing high quality knowledge graphs that could be used in clinical settings with minimal post-processing.

## Related work

In recent work, Finlayson *et al*. quantify the relatedness of 1 million concepts by computing their co-occurrence in free-text notes in the EMR, releasing a “graph of medicine”^22^. Sondhi *et al*. measure the distance between mentions of two concepts within a clinical note for determination of edge-strength in the resulting graph^23^. Goodwin *et al*. use natural language processing to incorporate the belief state of the physician for assertions in the medical record, which is complementary to and could be used together with our approach^24^. Importantly, whereas the aforementioned works consider purely associative relations between medical concepts, our methodology models more complex relationships, and our evaluation focuses on whether the proposed algorithms can derive known causal relations between diseases and symptoms.

## Methods

### Study design

We conducted a retrospective observational study using previously collected data from electronic medical records to construct a knowledge graph relating symptoms to diseases. We evaluated our candidate knowledge graphs against an extensive and manually curated knowledge graph provided by Google (Google health knowledge graph, or GHKG) and the expert opinion of physicians. The study was approved by our institutional review board.

### Setting and selection of participants

The study was performed using data from a 55 000-visit/year trauma center and tertiary academic teaching hospital. All consecutive emergency department (ED) patients between 2008 and 2013 were included. Each record represents a single patient visit. No patients were excluded, leading to a total of 273 174 records of emergency department patient visits.

### Data collection and preparation

#### Concept extraction from electronic medical record

We extracted positive mentions of diseases and symptoms (concepts) from structured and unstructured data. Structured data consisted of ICD-9 (International Classification of Diseases) diagnosis codes. Unstructured data consisted of chief complaint, Triage Assessment, Nursing Notes, and MD comments. Triage Assessment refers to the free-text nursing assessment documented at triage. Medical Doctor (MD) Comments and Nursing notes refer to the free-text scratch space used by physicians and nurses respectively to track a patient’s course. Free text fields were de-identified using PhysioNet’s deid software package^25, 26^.

**Figure 1:**
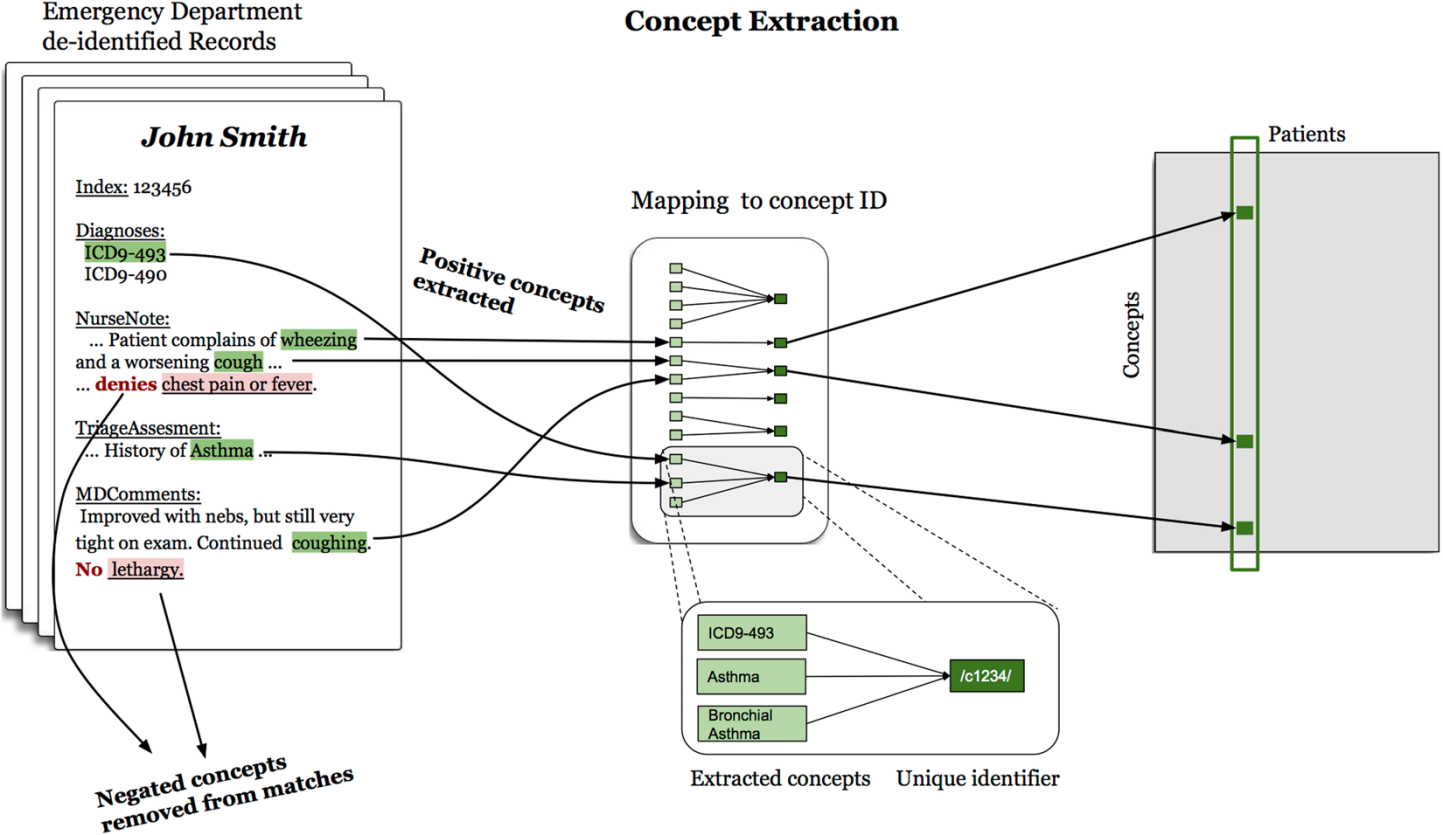
Concept extraction pipeline. Non-negated concepts and ICD-9 diagnosis codes are extracted from Emergency Department electronic medical records. Concepts, codes and concept aliases are mapped to unique IDs, which in turn populate a co-occurrence matrix of size (Concepts) × (Patients).

The set of diseases and symptoms considered were chosen from the GHKG (described below) to establish a basis for later comparison. We used string-matching to search for concepts via their common names, aliases or acronyms, where aliases and acronyms were obtained both from the GHKG as well as from the Unified Medical Language System (UMLS) for diseases where the mapping was known. Similarly, if a link to an ICD-9 code was provided, we searched for that code in the record’s structured administrative data. A modified version of NegEx was used to find negation scopes in the clinical text^27, 28^. Mentions that occured within a negation scope were not counted. Figure 1 illustrates the data extraction and processing pipeline.

#### Google health knowledge graph

A novel aspect of our study is the use of an expansive and manually curated health knowledge graph provided, with permission to use, by Google. The Google health knowledge graph, first announced in 2015, aims to empower users in their health decisions^19^. Google created the graph using a multi-step process that combined data mining techniques with extensive manual curation by a panel of experts. The graph is intended to be utilized by patients searching Google for health information (i.e., patient facing) and it is currently used within Google’s health panels that appear on the side of users’ screens in the US, Brazil and India in response to health-related search queries^29^.

We used a subset of the GHKG as of August 2015 for which we had sufficient support in our data. We defined sufficient support for a disease as having at least 100 positive mentions and for a symptom as having at least 10 positive mentions. This resulted in 156 diseases and 491 symptoms. The graph is comprised of medical concepts (diseases and symptoms) as nodes and disease-symptom relations as edges.

A small number of concepts in the GHKG are classified as both a disease and a symptom (e.g., ‘Type II diabetes’ is a disease, but also a symptom of ‘Polycystic Ovarian Cancer’). In these cases, we designated these concepts as diseases only.

Each concept included the common names for a concept, aliases and, when available, a mapping to ICD-9 codes and UMLS concepts. Additionally, a measure of a concept’s expected frequency is provided for both diseases and symptoms. For symptom nodes, the conditional expected frequency of a symptom given a disease’s presence is provided as either ‘frequent’ or ‘always’. For disease nodes, the frequency is described separately by age (‘senior’, ‘adult’, ‘young adult’, etc.) as ‘very frequent’, ‘frequent’, ‘rare’, ‘very rare’, or ‘never’.

### Algorithms for constructing a knowledge graph

**Figure 2:**
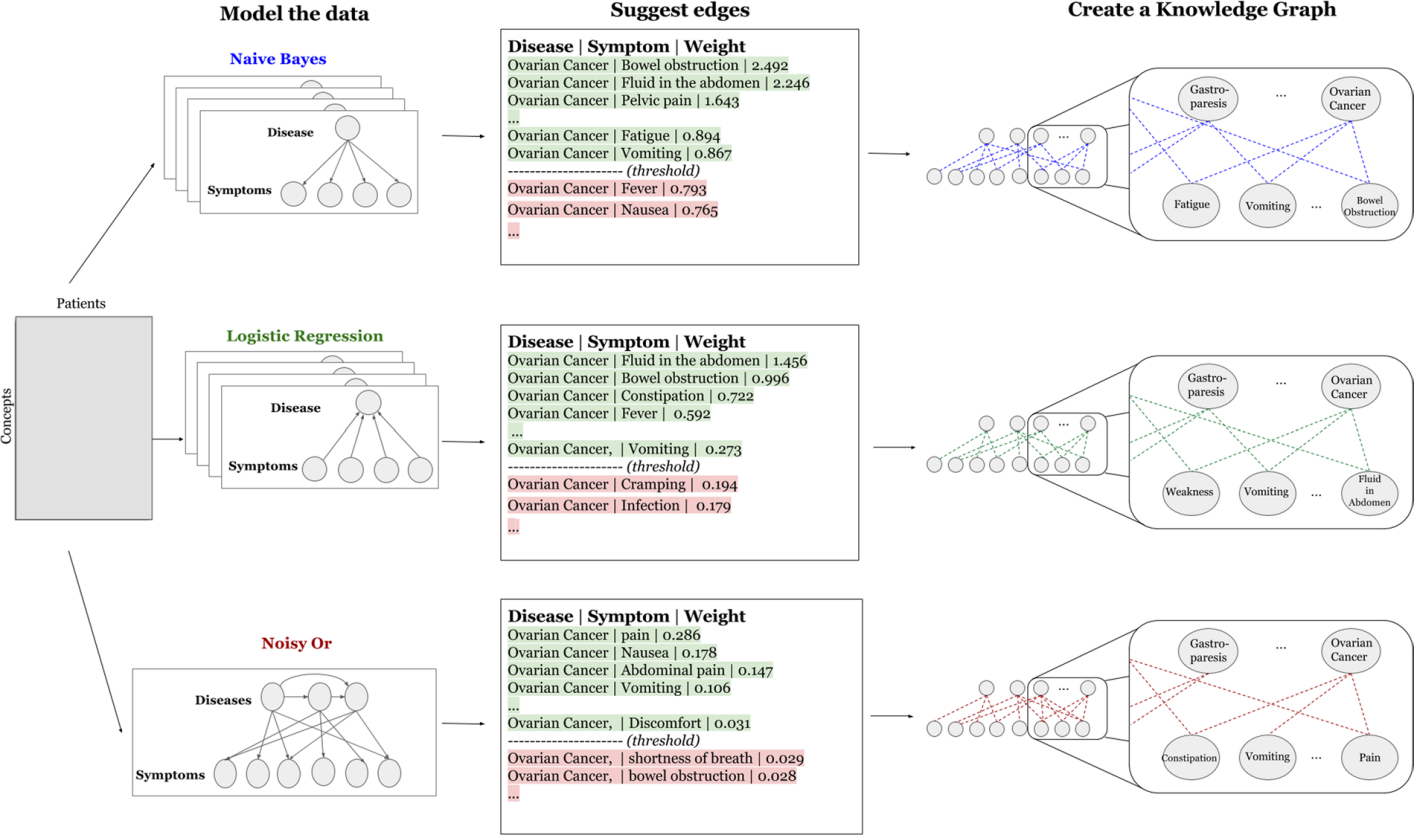
Workflow of modeling the relationship between diseases and symptoms and knowledge graph construction, for each of our 3 models (naive Bayes, logistic regression and noisy OR).

Learning the knowledge graph consists of three main steps. First, positive disease and symptom mentions were extracted from structured data and unstructured text (detailed in *‘Data collection and preparation’*). Second, statistical models relating diseases and symptoms were learned. Third, the learned statistical models were translated into knowledge graphs. The overall procedure is summarized in Fig. 2.

#### Parameter learning

We considered three statistical models: logistic regression (LR), naive Bayes (NB) and a Bayesian network modeling diseases and symptoms with noisy OR gates (noisy OR). Logistic regression, which is widely used for binary classification, was chosen as an example of a well-established machine learning classifier with interpretable parameters that is frequently used for modeling binary outcomes^30^. Naive Bayes was chosen as it provides a baseline of what can be inferred from simple pairwise co-occurrences^31^. Noisy OR was chosen as an example of a probabilistic model that jointly models diseases and symptoms; similar models have successfully been used in previous medical diagnosis applications^10, 12, 32–34^.

Parameters for all three models were learned using maximum likelihood estimation. For logistic regression and naive Bayes, a model was learned separately for each disease. For noisy OR, all the parameters were learned jointly. L1 regularization was used for logistic regression both to prevent overfitting and to encourage sparsity, which was desirable as we expect most diseases to cause only a small number of symptoms^35^. Laplacian smoothing was used to prevent overfitting for naive Bayes. For both, the values of the hyper-parameters were chosen separately for each disease via a 3-fold cross-validation.

#### Constructing the knowledge graphs

For each model, we construct an importance measure to determine whether an edge should be included between symptom and disease. The importance measures denote each model’s relative confidence that an edge exists between a pair of nodes. We then sort symptoms for each disease by the importance measure.

Logistic regression. The importance measure for logistic regression was taken to be:

1 $$IMP{T}_{LR}=Max({b}_{ij},0)$$

where ${{\rm{b}}}_{\mathrm{ij}}$ is the weight associated with symptom *i* in the logistic regression model fit to predict disease *j*. In other words, if the appearance of a symptom made a disease more likely, then we believed that a corresponding edge exists in the graph. Note that in our setting it is sensible to compare weights between features since all of our variables are binary.

Naive Bayes. The importance measure for naive Bayes was taken to be:

2 $$IMP{T}_{NB}=\,\mathrm{log}(p({x}_{i}=1|{y}_{j}=1))-\,\mathrm{log}(p({x}_{i}=1|{y}_{j}=0))$$

where ${{\rm{x}}}_{{\rm{i}}}$ is the binary variable denoting the presence of symptom *i* and y_j_ is the binary variable denoting the presence of disease *j*. We chose this as the importance measure because of its property that if the appearance of disease makes the observation of symptom more likely, we have higher confidence that an edge exists between the two. We further chose to focus on the multiplicative difference rather than the additive difference, as we wanted to capture the idea of increased relative risk. In other words, if a rare symptom became 3 times as likely due to the presence of a disease, but still remained rare, we want our model to suggest it as an edge.

For both naive Bayes and logistic regression, we enforced a minimum of 5 co-occurrences for any disease-symptom pair for any suggested edge as a de-noising measure.

Noisy OR. Noisy OR is a conditional probability distribution that describes the causal mechanisms by which parent nodes affect the states of children nodes. In our case, this pertains to mechanisms by which diseases affect the manifestation of its children symptoms. In a deterministic noise free setting, the presence of an underlying disease would always cause its symptoms to be observed, and a symptom could be observed if any of its parent diseases are ‘on’. For example, a patient would have a fever if they contracted the flu or if he/she has mononucleosis.

However, in real life the process is far less deterministic, which is where the “noisy” part comes in: a patient may not present with a fever even if he/she has the flu. Additionally, fever might occur as a result of neither flu nor mononucleosis. Noisy OR deals with the inherent noise in the process by introducing failure and leak probabilities. Specifically, a disease y_j_ that is present might fail to turn on its child symptom x_i_ with probability f_ij_. The leak probability l_i_ represents the probability of a symptom being on even if all of its parent diseases are off.

Thus the probability of a symptom being present is:

3 $$P({x}_{i}=1|{y}_{1},\mathrm{...},{y}_{n})=1-(1-{l}_{i}){\prod }_{j}{({f}_{ij})}^{{y}_{j}}$$

We took the importance measure to be:

4 $$IMP{T}_{noisy-or}=1-{f}_{ij}$$

We chose this as the importance measure because we wanted to express that higher importance means that the disease is more likely to turn on the corresponding symptom. Consider the case of diseases deterministically turning on symptoms. In this noise-free setting, the symptom fever would always be ‘on’ when flu is ‘on’, making the failure probability *f*_*ij*_ = 0 and our importance measure $IMP{T}_{noisy-or}=1$. This makes intuitive sense as we wish there to be an edge between fever and flu. In contrast, if a symptom never occurs for a disease, the corresponding failure probability would be 1 and the importance measure would be 0.

Importantly, by learning the model parameters using maximum likelihood estimation and deriving the importance measure from the conditional probability distributions, we make no assumptions about the prior distribution of diseases P(*y*_1_, …, *y*_*n*_). This is an important point that distinguishes noisy OR from logistic regression and naive Bayes, which implicitly assume that diseases are independent. Diseases are certainly not independent in the settings we consider. For example, given that patients tend to present with few diseases, the presence of one disease typically lowers the probability of others. Additional information on model assumptions is provided in the appendix.

### Analysis and Evaluation

We evaluate the quality of our constructed knowledge graphs by comparing them against the GHKG and expert physician opinion. We use a precision-recall curve to evaluate the graph structures derived from each measure.

Due to the resource intensive nature of the evaluation by physicians, we can only present a limited set of models to be evaluated by them. We chose to automatically evaluate our models against the GHKG in order to efficiently compare models at a negligible cost. We then select the two best performing models for evaluation by physicians.

#### Comparison to the Google health knowledge graph

The GHKG is a highly curated representation of knowledge compiled by several expert physicians via a multi step process. The labor-intensive curation of the graph results in a precise though not necessarily complete graph. Due to the non-exhaustive nature of the GHKG, this evaluation underestimates the precision of the models, marking edges as false-positives even when they may actually be correct and simply missing from the GHKG. Therefore, we do not take the automatic evaluation to be a true measure of model performance but rather consider it a relative measure against which to compare and rank the models.

To rank our models we assess their performance against a binary target (either the suggested edge is present in the GHKG or it is not). The symptom ‘pain’ was removed from this evaluation because it was overly general and inconsistently used in the GHKG.

Additionally, in the evaluation by physicians we evaluate the GHKG alongside our own models. By including the GHKG we are comparing our models against a painstakingly curated graph, which is widely used today via Google’s ‘health panels’, giving us a realistic benchmark for performance. Moreover, the inclusion of the GHKG allows us to validate our previous statement that GHKG is not complete by showing that our models surpass it in recall.

#### Evaluation by physicians

Given that the set of potential disease-symptom edges is large, it is impractical to ask evaluators to label all possible edges. Therefore, we use a procedure in which the top N results from each model are pooled together and rated by clinical evaluators. The edges outside the pooled results were considered irrelevant. This method, termed ‘pooling’, is frequently used in information retrieval settings^36^.

To evaluate the graphs, physicians rated the suggested edges according to the statement *“disease A causes symptom B”* using the 4-point scale: ‘always happens’, ‘sometimes happens’, ‘rarely happens’ or ‘never happens’. A user interface was built to facilitate easy tagging. Physicians were presented with the set of symptoms suggested by our top two models and the GHKG. Symptoms for tagging were presented in a random order to physicians, blinding them to the source of the suggestion. Further details of the clinical evaluation are provided in the appendix. For the evaluation itself, physician responses were binarized by grouping results from the ‘always’, ‘sometimes’ and ‘rarely’ categories into the positive category, leaving the ‘never’ tag to be negative. In the appendix we also present the results of the clinical evaluation with an alternative segmentation in which both ‘rarely’ and ‘never’ tags are assigned to the negative category.

#### Statistical methodology

In a paper surveying the reliability of pooling for evaluating the efficacy of information retrieval models, Zobel found that although the pooling method is overly optimistic in its evaluation of recall, it provides a reliable measure of precision and does not unjustly bias one method over another, providing a “fair basis of measurement of new systems”^37^. To determine whether the differences in model precision were statistically significant we use a Wilcoxon signed rank test, also suggested by Zobel^37^.

Due to the labor-intensive nature of the tagging process, only one physician tagged all diseases and symptoms. To determine generalizability of the tagging, a second physician tagged 15 randomly selected diseases and their corresponding symptoms and an inter-rater agreement measure was calculated. We use the spearman rho correlation to measure inter-rater agreement and calculate confidence intervals using bootstrapping.

### Data Sharing Statement

We provide the full knowledge graph learned using the noisy OR model in the appendix and in additional structured formats on the corresponding author’s website. The graph lists all 156 diseases and 491 symptoms, all edges between diseases and symptoms, and the importance scores associated with each edge.

The data set used in this study was derived from the electronic medical records from 273,174 patient visits to the Emergency Department at Beth Israel Deaconess Medical Center (BIDMC), and makes substantial use of free text notes written by clinicians that frequently include patient names, addresses, unique identifying numbers, birthdates, gender, rare diseases and treatments, socioeconomic status, workplace, number of pregnancies, and ethnicity. The BIDMC Institutional Review Board approved the usage of the data for this study, but precluded any public release of this data as it would violate patient privacy as specified by HIPAA as it contains protected health information. We, therefore, are not permitted to release this data set.

## Results

**Figure 3:**
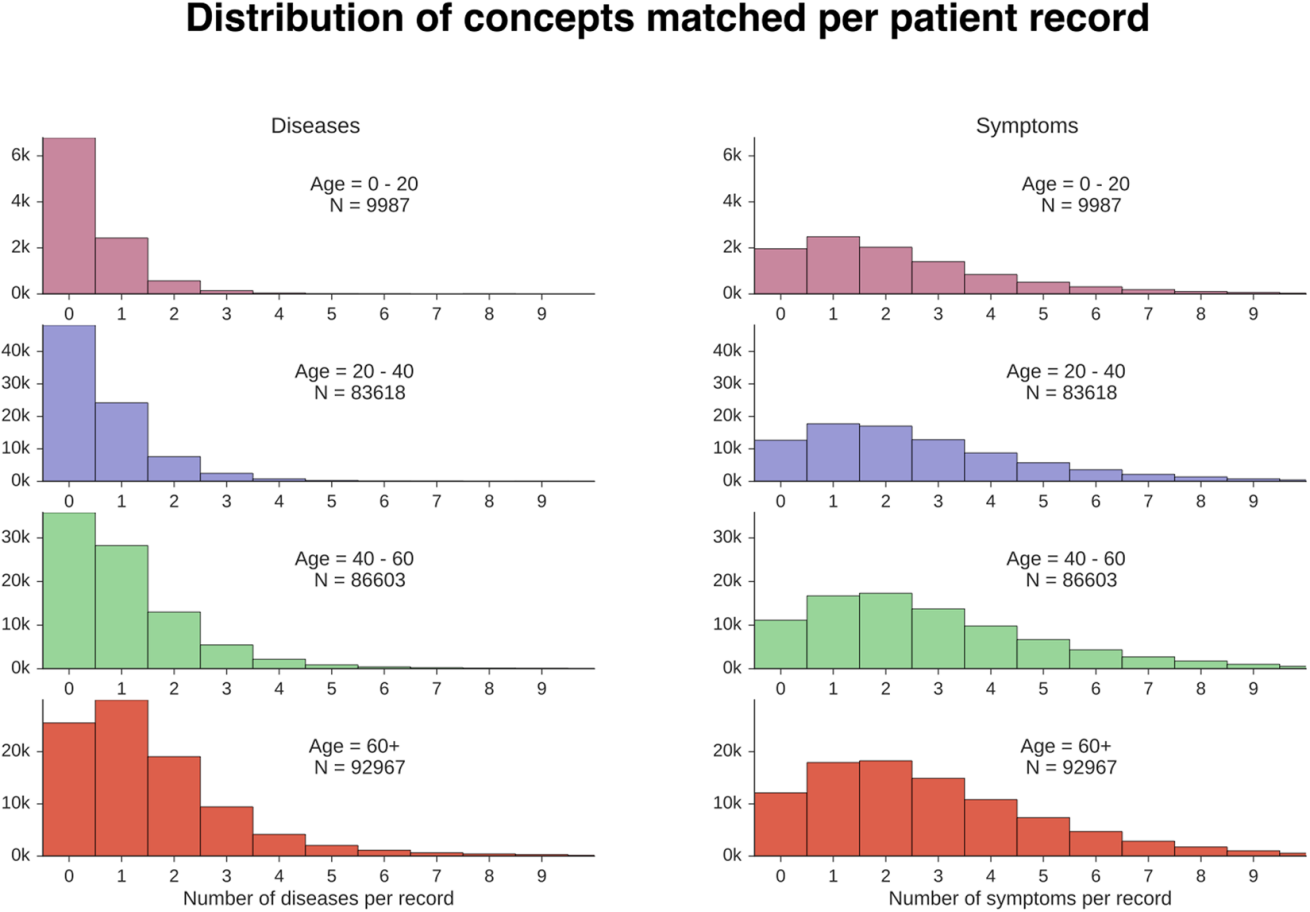
Distribution of number of diseases and symptoms per patient record.

Figure 3 shows the distribution of the number of identified diseases and symptoms across medical records. We observe that the distributions are positively skewed across age groups and that a substantial fraction of older patients (40+) have two or more diseases identified per record.

After concepts were extracted, we constructed a knowledge graph from each model by either choosing an importance threshold or by allowing each model to suggest a constant number of edges per diseases. Tables 1 and 2 showcase the top symptoms suggested by each model for ‘Middle Ear Infection’ and ‘Gallstones’, respectively. Both tables show that logistic regression is not well calibrated for the task of constructing a knowledge graph. In these examples, noisy OR and naive Bayes both perform similarly well.

**Table 1:** Top edge suggestions by models for a randomly chosen disease (Middle Ear Infection). The number of shown edges corresponds to the number of edges in the GHKG. For logistic regression, naive Bayes, and noisy OR the suggestions are ordered by their relative importance score. For the GHKG, the edges are sorted according to two broad buckets of edge frequency that are provided in the graph. The stars associated with each edge represent the expected frequency for which *“disease A causes symptom B”* as rated by physicians. [‘***’ = ‘always happens’, ‘**’ = ‘sometimes happens’, ‘*’ = ‘rarely happens’, ‘’ = ‘never happens’].

Top edge suggestions for ‘Middle Ear Infection’									
Ranking (importance score)	Logistic regression model		Naive Bayes model		Noisy OR model		Frequency (GHKG buckets)	GHKG	
**1**	Ear pain	***	Inflammation of ear	***	Ear pain	***	***Always***	Inflammation of ear	***
**2**	Teeth chattering		Ear pain	***	Inflammation of ear	***	***Frequent***	Ringing in the ears	**
**3**	Red face	*	Exudate	***	Sore throat	**	***Frequent***	Headache	**
**4**	Inflammation of ear	***	Ache	***	Coughing	*	***Frequent***	Nausea	*
**5**	Itchy eyes	**	Nasal congestion	*	Fever	**	***Frequent***	Crying	**
**6**	Irritability	**	Sore throat	**	Nasal congestion	*	***Frequent***	Fever	**
**7**	Anger	*	Runny nose	*	Pain	***	***Frequent***	Nasal congestion	*
**8**	Red rashes		Coughing	*	Ache	***	***Frequent***	Ear pain	***
**9**	Sleepiness	**	Sensitivity to light	*	Chills	**	***Frequent***	Loss of appetite	**
**10**	Facial paralysis		Fever	**	Headache	**	***Frequent***	Vertigo	*

**Table 2:** Top edge suggestions by models for ‘Gallstones’. The number of shown edges corresponds to the number of edges in the GHKG. For logistic regression, naive Bayes and noisy OR the edges are ranked by their relative importance score. For the GHKG, the edges are sorted according to two broad buckets symptom frequency that are provided in the graph [‘frequent’ and ‘always’]. The internal ordering of the edges within a given bucket is random. The stars associated with each edge represent the expected frequency for which *“disease A causes symptom B”* as rated by physicians. [‘***’ = ‘always happens’, ‘**’ = ‘sometimes happens’, ‘*’ = ‘rarely happens’, ‘’ = ‘never happens’].

Top edge suggestions for ‘Gallstones’									
Ranking (importance score)	Logistic regression Model		Naive Bayes Model		Noisy OR Model		Frequency (GHKG buckets)	GHKG	
**1**	Abdominal cramping from Gallstones	***	Abdominal cramping from Gallstones	***	Pain	***	***Frequent***	Back pain	**
**2**	Pain in upper-right abdomen	***	Pain in upper-right abdomen	***	Nausea	***	***Frequent***	Pain between shoulder blades	
**3**	Yellow skin and eyes	**	Upper abdominal pain	***	Abdominal pain	***	***Frequent***	Severe pain	***
**4**	Pain	***	Dark urine	*	Pain in upper abdomen	***	***Frequent***	Mild pain	**
**5**	Pain in upper abdomen	***	Yellow skin and eyes	**	Vomiting	***	***Frequent***	Night pain	
**6**	Dark urine	*	Pain in upper abdomen	***	Chills	*	***Frequent***	Abdominal discomfort	***
**7**	Upper abdominal pain	***	Intermittent abdominal pain	***	Tenderness	***	***Frequent***	Nausea	***
**8**	Dry skin		Belching		Abdominal cramping from Gallstones	***	***Frequent***	Side pain	*
**9**	Sleepiness	*	Discomfort in upper abdomen	***	Yellow skin and eyes	**	***Frequent***	Pain in upper-right abdomen	***
**10**	Abdominal pain	***	Abdominal pain	***	Pain in upper-right abdomen	***	***Frequent***	Flatulence	
**11**	Restless legs syndrome		Intermittent pain	***	Diarrhea	*	***Frequent***	Indigestion	*
**12**	Side pain	*	Swollen veins in the lower esophagus		Fever	**	***Frequent***	Vomiting	***
**13**	Regurgitation		Fluid in the abdomen		Flank pain	*	***Frequent***	Abdominal cramping from Gallstones	***

**Figure 4:**
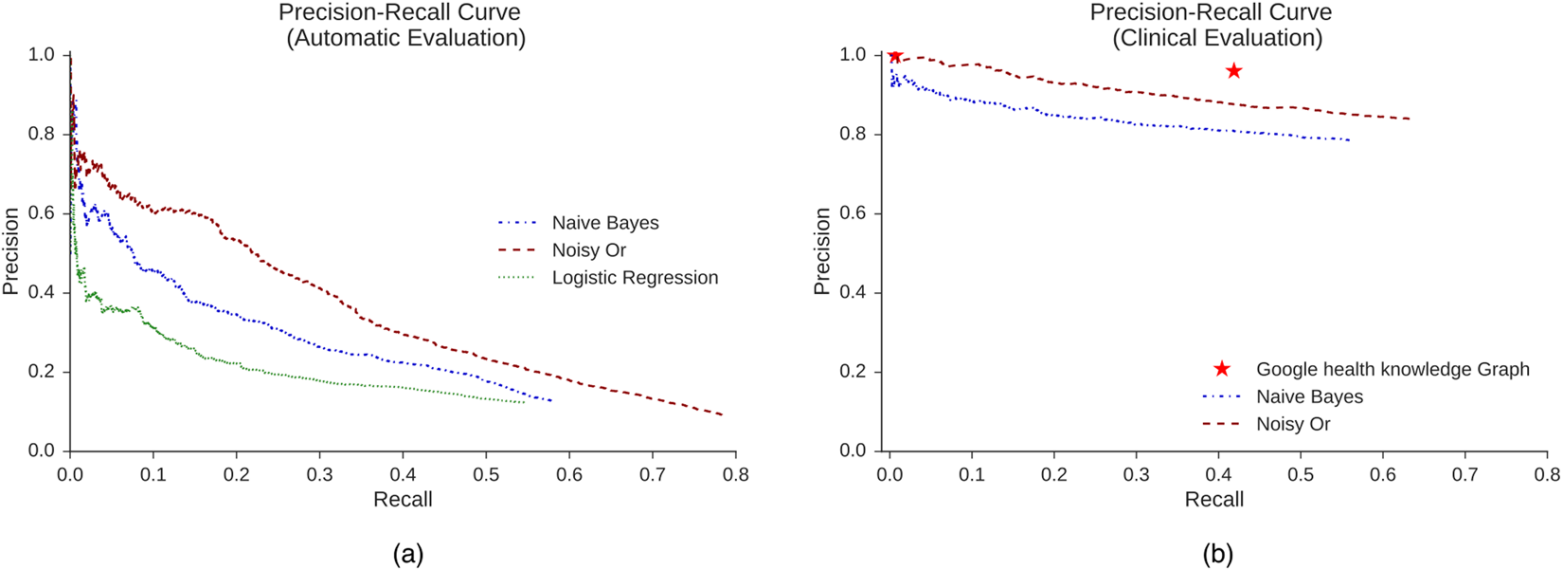
Precision-recall curves for two evaluation frameworks. (**a**) Precision-recall curve for the automatic evaluation evaluated against the GHKG. (**b**) Precision-recall curve rated according to physicians’ expert opinion. The red stars indicate thresholds corresponding to the two tags associated with symptoms in the GHKG (‘always’ or ‘frequent’). In both graphs, the relative performance of the models is the same.

The same trend is observed when evaluating precision-recall graphs across all available diseases. Figure 4(a) shows the Precision-Recall curve resulting from the automatic evaluation. Here too logistic regression falls short in performance. For instance, for a recall of 0.5, noisy OR, naive Bayes and logistic regression achieve a precision of 0.23, 0.18 and 0.13, respectively. As a result, we chose to eliminate logistic regression from the clinical evaluation. Figure 4(b) shows the Precision-Recall results for the clinical evaluation. The computed inter-rater agreement measure (*mean* = 0.7448, *std* = 0.0297) shows considerable agreement between evaluators, which give us confidence in the generalizability of the results. Additionally, we observe that both noisy OR and naive Bayes have lower recall and higher precision in the clinical evaluation than suggested by the automatic evaluation. For a recall of 0.5, noisy OR and naive Bayes achieve a precision of 0.87 and 0.8, respectively. The observation that both models surpass the recall of the GHKG in the clinical evaluation(Fig. 4(b)), suggests that our method is able to surface relevant symptoms that are not surfaced by the GHKG.

In both evaluation frameworks presented in Fig. 4, noisy OR outperforms naive Bayes. The Wilcoxon signed rank test determined that the differences in precision were statistically significant for both evaluation frameworks (p<=0.01).

Table 3 shows a subset of the knowledge graph constructed by noisy OR, our best performing model (for full graph, see appendix). The number of edges included was chosen to match the number of symptoms presented to clinical evaluators (see appendix).

**Table 3:** Subset of the knowledge graph learned using the noisy OR model. For each disease we show the full list of edges along with their corresponding importance score in parentheses. Symptoms are ordered according to importance scores.

Examples of Edge Suggestions for Noisy OR	
**Diseases**	Suggested edges
Aphasia	problems with coordination (0.318), weakness (0.181), confusion (0.106), mental confusion (0.088), slurred speech (0.074), numbness (0.071), headache (0.049), seizures (0.045), weakness of one side of the body (0.042), difficulty speaking (0.034), blurred vision (0.018), malnutrition (0.017)
Appendicitis	pain (0.881), nausea (0.401), abdominal pain (0.361), tenderness (0.163), chills (0.152), diarrhea (0.124), vomiting (0.118), fever (0.096), loss of appetite (0.068), lower abdominal pain (0.040), cramping (0.037), constipation (0.036), discomfort (0.033), cyst (0.030), pain in right lower abdomen (0.029), sharp pain (0.023), pain during urination (0.022), pain in upper abdomen (0.020), pelvic pain (0.017), flank pain (0.016), vaginal discharge (0.013), abdominal discomfort (0.013), dull pain (0.012), infection (0.011)
Bed bug bite	skin rash (0.329), itching (0.173), anxiety (0.048), infection (0.029), sadness (0.026), depression (0.026), red spots (0.018), skin irritation (0.018), sweating (0.016), eye pain (0.015), lesion (0.012), substance abuse (0.011), hallucination (0.009), swollen feet (0.009), skin lesion (0.009), brief visual or sensory abnormality (0.009)
Bell’s palsy	numbness (0.308), weakness (0.198), headache (0.134), facial paralysis (0.071), ear pain (0.052), slurred speech (0.051), paralysis (0.046), facial pain (0.040), neck pain (0.038), facial swelling (0.037), tongue numbness (0.031), asymmetry (0.026), blurred vision (0.024), drooping of upper eyelid (0.020), lesion (0.019), malnutrition (0.019), difficulty swallowing (0.018), double vision (0.016)
Carpal tunnel syndrome	numbness (0.175), pain (0.167), hand pain (0.094), weakness (0.083), arm pain (0.071), wrist pain (0.060), swelling (0.054), hand numbness (0.041), redness (0.030), pins and needles (0.024), shoulder pain (0.024), vertigo (0.020), hand swelling (0.016), neck pain (0.016), infection (0.014), depression (0.011), sadness (0.011), anxiety (0.011), chronic back pain (0.010), back pain (0.010), malnutrition (0.010), severe pain (0.008), unsteadiness (0.008), dry skin (0.008)
Ectopic pregnancy	pain (0.537), bleeding (0.204), vaginal bleeding (0.181), abdominal pain (0.167), cramping (0.155), spotting (0.154), nausea (0.104), cyst (0.067), tenderness (0.055), lower abdominal pain (0.048), pelvic pain (0.040), diarrhea (0.031), vaginal discharge (0.023), discomfort (0.020), vomiting (0.016), back pain (0.015), vaginal pain (0.014), lightheadedness (0.011)
Kidney stone	pain (0.608), flank pain (0.495), nausea (0.232), blood in urine (0.141), pain during urination (0.084), vomiting (0.083), chills (0.067), abdominal pain (0.065), back pain (0.050), tenderness (0.040), discomfort (0.019), groin pain (0.018), severe pain (0.013), fever (0.012), testicle pain (0.011), frequent urge to urinate (0.011), lower abdominal pain (0.011), dark urine (0.011), urinary retention (0.011), sharp pain (0.010), cyst (0.010), pain in lower abdomen (0.010), diarrhea (0.009), constipation (0.008), infection (0.007), pelvic pain (0.007), side pain (0.004), dull pain (0.004)
Retinal detachment	vision loss (0.125), blurred vision (0.065), headache (0.057), neck pain (0.041), eye pain (0.039), dehydration (0.024), difficulty walking (0.023), itching (0.020), discomfort (0.018), unequal pupils (0.017), watery diarrhea (0.015), bone loss (0.015), partial loss of vision (0.014), ear pain (0.013), fast heart rate (0.012), slow bodily movement (0.009), low oxygen in the body (0.009), vision disorder (0.009), elevated alkaline phosphatase (0.009), seeing spots (0.009), abnormality walking (0.009), malnutrition (0.009)

## Discussion

There were a number of differences between the edges suggested by the learned models and marked as correct by the clinical evaluators and those contained in the Google health knowledge graph. For one, the GHKG was designed to provide useful information to web-users, which explains some of the differences between it and the emergency department setting where the data was collected. As a result of its patient-facing design, the GHKG is not exhaustive. Some examples of omissions from the GHKG for the disease ‘Middle Ear Infection’ include ‘Exudate’, ‘Ache’ and ‘Sore throat’, which were labeled as highly relevant by both of our clinical evaluators (Table 1). Similarly, the symptoms ‘Tenderness’ and ‘Intermittent pain’ are not listed in the GHKG’s symptoms for the disease ‘Gallstones’ (Table 2). These symptoms were suggested by our learning algorithms, illustrating the potential for an EMR data-driven approach to uncover relevant symptoms.

Additionally, the higher recall achieved by our model shows that it is surfacing relevant symptoms that are not surfaced by the GHKG. These additional symptoms typically include infrequent symptoms that are not easily elicited from doctors, but are still medically and diagnostically relevant. Examples of edges that are suggested by our models, not present in the GHKG, and tagged by our clinical evaluators as ‘rarely happens’ include: ‘Dizziness’ as a symptom of ‘Type 2 Diabetes’, ‘Heartburn’ and ‘Dark urine’ as symptoms of ‘Gallstones’ and ‘Rectal pain’ as a symptom of ‘Prostate Cancer’, just to name a few.

Another class of differences between edges approved by the clinical evaluators and edges in the GHKG involves the preciseness of language used. For example, the GHKG contains an edge from ‘Gallstones’ to ‘Pain between shoulder blades’. While this is technically not the precise location of gallstone pain, it is a description that a patient may use.

**Figure 5:**
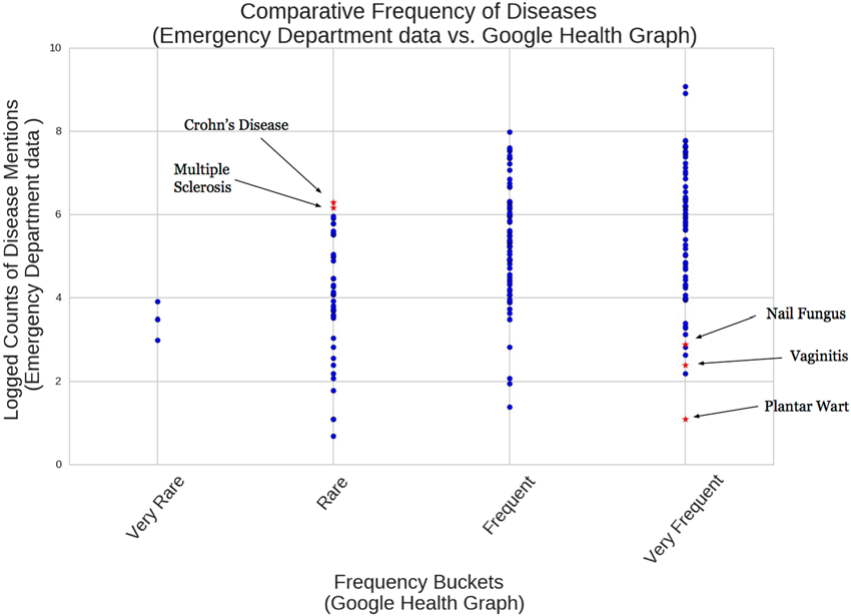
Comparison of disease frequency for the ‘adult’ age bracket (40–60 years old). The y-axis shows the number of identified diseases in the emergency department data. The x-axis records the expected frequency of diseases according to the Google health knowledge graph for the ‘adult’ age bracket. The points highlighted demonstrate instances of frequency misalignment due to the differences in populations considered.

A third class of differences involves a heightened severity of the edges suggested by our models. For instance, for the disease ‘Gallstones’ (Table 2), the clinical collaborators approved ‘Abdominal Pain’, while the GHKG only contains the edge ‘Abdominal Discomfort’. Similarly, our models suggest ‘Diarrhea’ in place of the milder ‘Indigestion’. Our model’s selection of more severe presentations of edges suggests that the graph is organically tailored for the emergency department data setting. Figure 5 shows the expected frequency of diseases, as listed in the GHKG for the ‘adult’ age bracket, compared to the observed count of diseases for that age bracket as found in our data. Both ‘Multiple Sclerosis’ and ‘Crohn’s Disease’ appear very frequently in the emergency department data even though they are listed as ‘Rare’ in the GHKG. Conversely, ‘Vaginitis, ‘Plantar Wart’ and ‘Nail Fungus’ appear very infrequently in the emergency department data, even though they are listed as ‘Very Frequent’ according to the GHKG. This selection bias towards higher acuity conditions and presentations leads to structural differences between our constructed graphs and the GHKG, and suggests that our methodology provides a way of automatically adapting a knowledge graph across a range of different settings.

Next, we look at the most common symptoms that are wrongly suggested by each model to determine if there are certain characteristic errors that our learning algorithms make.

The noisy OR model tends to rank general symptoms highly, such as ‘Pain’, ‘Weakness’, ‘Lethargy’, ’Sadness’ and ‘Infection’. For example, in Table 2 we see that for disease ‘Gallstones’ noisy OR suggests ‘Pain’, ‘Nausea’ and ‘Abdominal Pain’ before the more specific symptom ‘Abdominal Cramping from Gallstones’. While these edge suggestions are not necessarily incorrect, they are substantially less informative than their more specific counterparts. This trend is not shared by the naive Bayes and logistic regression models.

Both naive Bayes and logistic regression wrongly suggest symptoms that are highly correlated with confounding factors and are not necessarily relevant to the parent disease. For instance, both models suggest ‘Bone Loss’, ‘Lethargy’ and ‘Confusion’ as likely symptoms for diseases that are common in elderly patients. For example, ‘Bone Loss’ is weighted highly for diseases such as ‘Shingles’ and ‘Hiatal Hernia’. ‘Lethargy’ is weighted highly for ‘Thyroid cancer’, ‘Myasthenia Gravis’ and ‘Neutropenia’. These incorrect edges are likely being suggested because old age is a confounding factor that results in these diseases being correlated with one another. The problem of disambiguating correlation and causation is partly avoided using the noisy OR model. We give a formal explanation for this in the appendix. We note that this is particularly relevant since many patients have multiple diseases (Fig. 3).

### Limitations

Our evaluation focused on the ability of the proposed algorithms to recover known causal relations involving diseases and symptoms. However, any approach that seeks to infer causal relations from observational data, as we do, inherently has major limitations. For example, unobserved confounding factors will affect the ability of all of the proposed approaches to infer correct causal relations^38, 39^, and proving causality would require many additional experiments. Rather, our algorithms should be construed as only providing candidate causal relations.

The precision and recall obtained by our constructed knowledge graphs show that reasonable results can be obtained even with a rudimentary concept extraction pipeline. Nonetheless, because of the simplicity of the pipeline, at times we do not have coverage for concepts despite them being present in the emergency department data. More precisely, 34% of the symptoms from the GHKG did not reach the required threshold of 10 positive mentions and were dropped due to insufficient support. One example is the symptom ‘Bull’s Eye Rash’ for disease ‘Lyme Disease’. Because of the varying ways in which the symptom is recorded and punctuated (for example: “bullseye”, “bullseye rash”, “bull eye”, “bull’s eye”, etc.), we record it fewer than 10 times. A more elaborate concept extraction pipeline would increase our coverage and improve the subsequent graph.

While our pipeline does not require any prior knowledge of the target area of application, it does require a base set of concepts to evaluate as potential nodes in the graph. For evaluation purposes, we used the concepts from the GHKG. For alternate uses, any set of concepts, such as UMLS, would be appropriate. Nonetheless, it is important to recognize that our task was made simpler by working with a set of concepts clearly delineated into ‘diseases’ and ‘symptoms’ (UMLS’s classification can be inconsistent) and for which every symptom is relevant to at least one disease.

Another limitation of our study is the underlying modeling assumptions inherent in our models. Neither noisy OR nor our baseline models allow for edges to exist between the symptom nodes. It may be reasonable to allow symptoms to cause other symptoms as well, creating a softer classification into symptoms and diseases. An example where a softer classification might be useful is in the previously mentioned ‘Type II diabetes’, which is a symptom of ‘Polycystic Ovarian Cancer’, but may itself cause other symptoms such as ‘Fatigue’ or ‘Frequent Urination’. Future research might benefit from investigating models that do not assume symptom conditional independence in order to capture this complexity.

Lastly, all the models we have applied to the problem of knowledge graph construction are parametric and therefore restricted by their parametric form (e.g., noisy OR conditional distributions). It might be useful to look into models that are not constrained by this form, particularly in order to have a closer match with the causal interpretation presented in the appendix.

## Conclusions

We find that it is possible to construct a high quality health knowledge graph directly from electronic medical records. The high precision displayed by the noisy OR model with a precision of 0.85 for a recall of 0.6 suggests that a two-step process would be well suited for the construction of the knowledge graph, in which a clinician reviews and rejects some of the edges suggested by the model. Using the results of the clinical evaluation, we can infer that if a filtering step were added to the pipeline, to achieve perfect precision with a corresponding recall of 60%, physicians would have to discard fewer than 2 out of 10 suggested edges. If this step were added to the pipeline, the resulting graph would have perfect precision and recall that would far exceed that of the Google health knowledge graph, making it an attractive candidate for real life applications. This prospect is made all the more attractive by our model’s ability to surface ‘rare’ symptoms that are not easily elicited from doctors. This “clean up” phase is also used in other approaches for constructing knowledge bases: since text mining and natural language processing are typically imperfect^40^, state of the art methods still use some degree of manual checking by physicians to ensure the quality of the compiled knowledge bases^19, 41^.

This method of automatically constructing knowledge graphs allows us to create graphs from EMRs in any number of domains quickly and without any prior knowledge. We believe that the most promising avenues for future research include incorporating more elaborate concept extraction algorithms into our pipeline and experimenting with other methods of measuring causal effects that do not assume a parametric form.

In addition to creating new knowledge graphs, such automated algorithms can be used to augment and maintain existing knowledge graphs. For example, they can be run regularly on current EMR data with existing knowledge graphs to suggest new edges over time that were not previously known. They can also be used to calibrate a knowledge base created for one setting to an entirely different setting.

## Electronic supplementary material

Supplementary Information
