# Supplementary material for: Learning a Health Knowledge Graph from Electronic Medical Records
Source: Sci Rep. 2017 Jul 20;7:5994. doi: 10.1038/s41598-017-05778-z (PMC5519723; doi:10.1038/s41598-017-05778-z)
Supplement: Supplementary file 1 — Supplementary Information [file 41598_2017_5778_MOESM1_ESM.pdf]

Supplementary Information - Learning a Health Knowledge Graph from Electronic Medical Records

Maya Rotmensch[1]; Yoni Halpern, PhD[2]; Abdulhakim Tlimat, MD[3]; Steven Horng, MD MMSc[3]; David Sontag, PhD[4ǂ]

[1] Center for Data Science, New York University, New York, NY. USA

[2] Department of Computer Science, New York University, New York, NY. USA

[3] Department of Emergency Medicine, Beth Israel Deaconess Medical Center, Boston, MA. USA

[4] Department of Electrical Engineering and Computer Science, Massachusetts Institute of Technology, Cambridge, MA. USA

Appendix 1: Methodology Details

Model Assumptions

Below we review some of the main assumptions that have biased our models and shaped their performance.

The defining assumption for naïve Bayes is of course its assumption of conditional independence between child nodes given the observation of the parent node. In our context this translates to symptoms being conditionally independent from one another given the parent disease. This is an over simplification, as is it possible that for a given disease, the appearance of one symptom makes another symptom more likely. For instance, for the disease Bronchitis, both congestion and headache are common symptoms. While both do not always occur when the disease is present, the presence of congestion raises the likelihood of a headache.

While Logistic Regression does not assume conditional independence between symptoms, it does rely on limited multicollinearity to perform well. Feature selection and regularization are common ways of limiting the performance issues associated with multi multicollinearity. In our paper we have opted to perform an extensive grid search of the proper regularization parameter to mitigate this problem. However, as we describe in the section below, optimizing a criteria focused on predicting disease from symptoms may be mismatched to the goal of learning a good health knowledge graph.

Additionally, for naïve Bayes and logistic regression each disease is modeled separately, resulting in an assumption of independence between diseases. One of the main advantages of the application of the noisy OR model in this setting is that it does not make any assumptions about the prior distribution of diseases $P(y_1, \ldots, y_n)$. This assumption is extremely important for our application, where many patients present with more than one disease (see Figure 3).

A central assumption made by the noisy OR model is the assumption of independence of effects. This assumption is an oversimplification, as the presentation of symptoms in a co-morbid scenario could very different than the presentation each disease produces separately. Nonetheless, a recent paper by Anand and Downs, which reviews the implication of this assumption on model performance, found that a model constructed with the noisy OR assumption "performs comparably" to a Bayesian network where no restriction were placed on the conditional probability distribution. The authors go on to claim that, at least for the setting that they consider, the minor differences that do exist between the two are "unlikely to be clinically important".[1]

Modeling Details

A threshold of at least 5 co-occurrences between disease and symptom pairs was chosen as a denoising measure for naïve Bayes and Logistic Regression, in order to allow us to compute the necessary statistic with some confidence. We chose a small number so as to not unfairly bias against rare diseases. While we did not do an exhaustive grid search, we experimented with several co-occurrence thresholds (0,2,5,10) and chose the smallest value that performed well on the automatic evaluation

Evaluation by physicians

Given the resource intensive nature of the physician evaluation, we use a procedure in which the top N results from each model are pooled together and rated by clinical evaluators. For a given disease, the top two models from the automated evaluation, as well as the industry-provided graph, contributed to the pool of edges to be evaluated by the physicians. Each model suggests up to K+10 symptoms, where K is the number of symptoms present in the knowledge graph for that disease. The 10 additional symptoms are introduced to allow for the possibility of the true graph being denser than the Google health knowledge graph. The figure below shows the user-interface though which the physicians tagged edges.

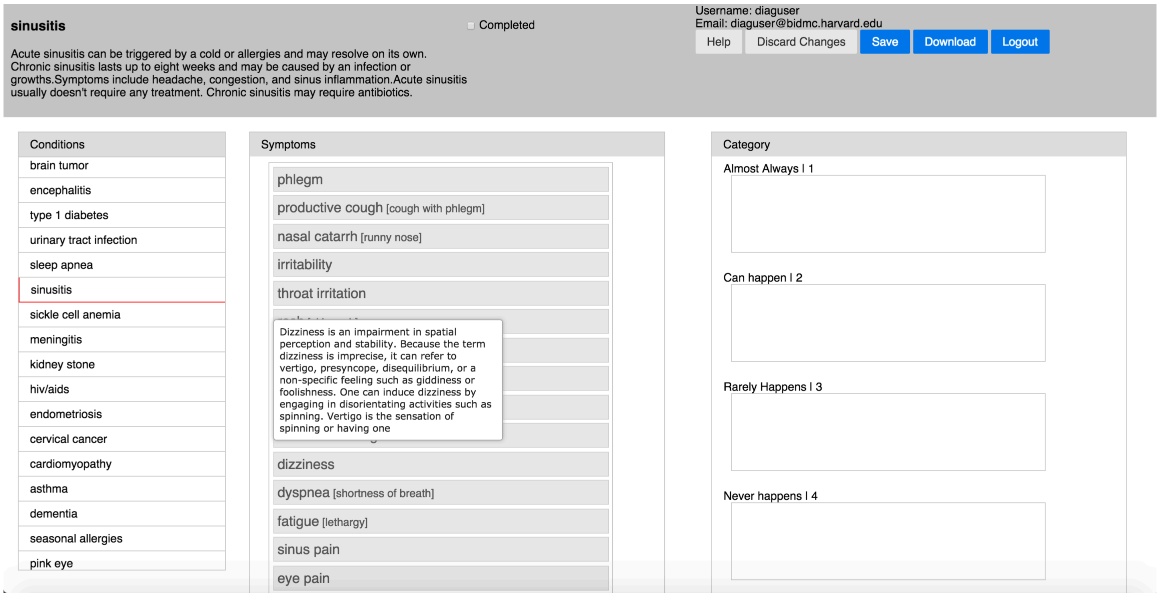

Figure 1: user interface for evaluation by physicians. The left column shows the list of diseases from which clinicians select a disease to be tagged. When a disease is selected, the upper panel provides a more detailed description of the selected disease. The middle column shows the symptoms that have yet to be tagged, and hover over functionality provides a detailed symptom definition. Lastly, the rightmost column shows the 4 categories into which symptoms can be sorted.

Statistical Significance

The Wilcoxon signed ranked test was used to determine the statistical significance of the differences in precision as it is non paramedic and does not assume the population is normally distributed. In the test, each pair of points represent the precision for a given condition by models a and b, where a and b represent our two best performing models. For both the automatic and clinical frameworks, model precision was calculated for the top K+10 symptoms suggested by each model.

GHKG's Precision-Recall

In the clinical evaluation we compare our resulting graphs against expert physician opinion using a precision-recall curve. We evaluate the Google knowledge health graph alongside our own models as a way to provide a realistic benchmark for performance. The GKHG does not contain continuous edge weights that are directly comparable to our generated importance measures. However, each edge in the graph is tagged with a frequency measure of 'frequent' or 'always' to indicate how often the symptom appears given the presence of the disease. To evaluate the GHKG against our model, we used the frequency tags as a measure of confidence in the existence of an edge. The star on the left represents the GHKG's performance for the 'high confidence' setting and includes only edges that were marked as 'always' in the graph. The star on the right represents the graph's performance in the setting in which it was allowed to suggest all edges that were deemed relevant, regardless of whether they occurred 'always' or 'frequently'.

A causal explanation for model performance

In reasoning broadly about the differences in model performance, we recall that the knowledge graph is, in its essence, a causal graph describing how diseases cause symptoms. In what follows we give a formal framework for characterizing what might make a good knowledge graph, and demonstrate that our results are consistent with what one would expect under this framework.

The casual query that we conjecture defines a good knowledge graph can be formalized via Pearl's 'do' operator [2] in which we intervene and set a disease to be either on or off, and see how this affects the likelihood of a symptom:

$$IMPT_{causal} = \frac{P(x_i = 1 | do(y_j = 1))}{P(x_i = 1 | do(y_j = 0))} \quad (5)$$

In other words, we believe that the weight of an edge i, j in the true graph is closely tied to the likelihood ratio of the symptom x_i manifesting, given that we *intervene* to set a disease y_j to be either on or off. By formulating the importance measure using Pearl's do-calculus, we are able to disentangle correlations between a disease and a symptom that occur simply because the disease frequently occurs with other diseases that cause the symptom.

This target quantity (Equation 5) is not aligned with the objective optimized by logistic regression, which explicitly models $P(y_j | X)$. This misalignment may explain logistic regression's consistently poor performance.

Naive Bayes, which consistently performs better than logistic regression and worse than noisy OR, uses the logarithm of the formula shown in Equation 5 as its importance measure, but assumes that the interventional distribution $P(x_i = 1 \mid do(y_j))$ is equal to the conditional distribution $P(x_i | y_j)$. However, one can show that these two are equal *only if the parent diseases are marginally independent*. To the extent that diseases are not independent, the estimate of the edge strengths provided by naive Bayes will be biased.

In contrast, the measure of edge importance used by noisy OR can be shown to correspond to a causal quantity closely related to the target quantity shown in Equation 5, specifically the do likelihood ratio of the symptom being *off* instead of on:

$$IMPT_{noisy-or_{ij}} = 1 - \frac{P(x_i = 0 | do(y_j = 1))}{P(x_i = 0 | do(y_j = 0))} \quad (6)$$

Moreover, the learning method used by noisy OR, which optimizes the conditional likelihood of the observed symptoms given the diseases, makes no assumptions about the prior distribution of the diseases. In conclusion, we believe that this close alignment explains noisy OR's significantly better performance in our evaluations.

Appendix 2: The Constructed Graph

Below is the full graph generated by the noisy or model with a threshold of K+10 edges, where K is the number of edges in the Google health knowledge graph for the corresponding condition. These are precisely the same edges for noisy or rated by the physicians in the clinical evaluation and displayed in Figure 3(b). The graph presented corresponds to a recall of 0.63 and a precision of 0.84.

**Knowledge graph**<br>(Generated by the noisy or model)	
Diseases	Symptoms
abscess	pain (0.318), fever (0.119), swelling (0.112), redness (0.094), chills (0.092), infection (0.083), cyst (0.047), tenderness (0.037), rectal pain (0.026), lesion (0.025), lump (0.023), sore throat (0.021), facial swelling (0.016), pimple (0.016), discomfort (0.014), difficulty swallowing (0.013), cavity (0.013), night sweats (0.007), severe pain (0.007), abdominal pain (0.007), painful swallowing (0.007), back pain (0.006)
acid reflux	pain (0.225), nausea (0.140), pain in upper abdomen (0.064), abdominal pain (0.058), sadness (0.052), depression (0.052), vomiting (0.048), anxiety (0.045), diarrhea (0.044), discomfort (0.034), indigestion (0.030), chills (0.022), constipation (0.021), dizziness (0.021), heartburn (0.020), chest pressure (0.017), coughing (0.015), difficulty swallowing (0.014), tenderness (0.014), lightheadedness (0.013), shortness of breath (0.012), belching (0.012), racing heartbeat (0.011), chest discomfort (0.011), burning sensation (0.011), discomfort in upper abdomen (0.010), chest tightness (0.009), fatigue (0.009), cramping (0.009), lethargy (0.008), headache (0.006), sharp pain (0.006), lump (0.005), dry cough (0.005), abdominal discomfort (0.005), malnutrition (0.005), weight loss (0.005), bone loss (0.004)
acute renal failure	kidney failure (0.127), weakness (0.087), diarrhea (0.062), lethargy (0.058), low blood pressure (0.053), dehydration (0.048), vomiting (0.044), nausea (0.041), fatigue (0.033), confusion (0.029), mental confusion (0.022), dizziness (0.021), malaise (0.014), weight loss (0.012), urinary retention (0.010), shortness of breath (0.009), blood in urine (0.009)
alcohol intoxication	slurred speech (0.071), vomiting (0.064), unsteady gait (0.056), depression (0.034), sadness (0.034), confusion (0.029), falling (0.025), thoughts of suicide (0.019), wandering and getting lost (0.018), agitation (0.017), crying (0.015), screaming (0.015), seizures (0.013), fainting (0.013), bruising (0.010), substance abuse (0.007), tremor (0.005), rapid involuntary eye movement (0.005), difficulty walking (0.005), snoring (0.005), fast heart rate (0.004), problems with coordination (0.004), leaking of urine (0.002), hunger (0.002), low oxygen in the body (0.002), aggression (0.002)
alcoholism	seizures (0.125), depression (0.090), sadness (0.090), slurred speech (0.061), anxiety (0.048), unsteady gait (0.030), fast heart rate (0.028), tremor (0.027), thoughts of suicide (0.023), agitation (0.016), substance abuse (0.015), confusion (0.012), visual hallucinations (0.012), shakiness (0.011), falling (0.009), vomiting (0.008), headache (0.008), screaming (0.007), vomiting blood (0.006), hearing voices (0.005), rapid involuntary eye movement (0.004), delirium (0.004), sweating (0.004), leg pain (0.004), wandering and getting lost (0.004), mental confusion (0.004)
alzheimer's disease	confusion (0.071), mental confusion (0.057), fainting (0.028), lethargy (0.026), weakness (0.025), agitation (0.025), bruising (0.018), depression (0.014), sadness (0.014), leaking of urine (0.014), falling (0.013), seizures (0.010), hip pain (0.010), unsteady gait (0.009), aggression (0.007), sleepiness (0.007), leg pain (0.007), wandering and getting lost (0.007), failure to thrive (0.006), drooping of upper eyelid (0.006), memory loss (0.006), slow heart rate (0.006), malnutrition (0.005), coughing (0.005), weight loss (0.005), low blood pressure (0.005), abnormality walking (0.005)

anaphylaxis	swelling (0.154), shortness of breath (0.099), itching (0.084), wheezing (0.082), redness (0.076), skin rash (0.072), facial swelling (0.053), tongue swelling (0.048), difficulty breathing (0.044), swelling under the skin (0.044), difficulty swallowing (0.044), anxiety (0.036), noisy breathing (0.032), chest tightness (0.029), diarrhea (0.029), fainting (0.024), nausea (0.024), fast heart rate (0.020), seizures (0.020), difficulty speaking (0.016), abdominal pain (0.014), flushing (0.014), crying (0.014), respiratory distress (0.013), hoarseness (0.013), itchy eyes (0.013), low blood pressure (0.013), acute episodes (0.012), infection (0.011), fatigue (0.010), dry mouth (0.010), chest discomfort (0.010), flank pain (0.009), joint pain (0.008), malnutrition (0.008), impaired voice (0.008)
anemia	lethargy (0.096), weakness (0.087), fatigue (0.086), shortness of breath (0.052), dizziness (0.047), bleeding (0.046), lightheadedness (0.027), malnutrition (0.025), nausea (0.024), sadness (0.024), diarrhea (0.024), depression (0.024), fainting (0.020), low blood pressure (0.020), constipation (0.019), infection (0.017), abdominal pain (0.014), kidney failure (0.014), dehydration (0.013), vaginal bleeding (0.011), dark stool from digested blood (0.010), racing heartbeat (0.010)
aneurysm	headache (0.128), dizziness (0.047), pain (0.047), nausea (0.046), weakness (0.037), back pain (0.028), neck pain (0.020), vertigo (0.018), fainting (0.016), numbness (0.015), blurred vision (0.014), lightheadedness (0.014), severe headache (0.013), seizures (0.011), ache (0.011), drooping of upper eyelid (0.009)
angina	pain (0.352), shortness of breath (0.154), chest pressure (0.077), nausea (0.075), coughing (0.060), chest discomfort (0.041), racing heartbeat (0.039), chest tightness (0.035), dizziness (0.031), anxiety (0.029), lightheadedness (0.022), headache (0.021), discomfort (0.021), back pain (0.020), arm pain (0.020), cough with phlegm (0.015), numbness (0.014), chest pain worsened by breathing (0.014), fatigue (0.013), depression (0.012), fast heart rate (0.012), sadness (0.012), sharp pain (0.012), pain in upper abdomen (0.012), lethargy (0.011), indigestion (0.010), fainting (0.010), chills (0.009)
aphasia	problems with coordination (0.318), weakness (0.181), confusion (0.106), mental confusion (0.088), slurred speech (0.074), numbness (0.071), headache (0.049), seizures (0.045), weakness of one side of the body (0.042), difficulty speaking (0.034), blurred vision (0.018), malnutrition (0.017)
appendicitis	pain (0.881), nausea (0.401), abdominal pain (0.361), tenderness (0.163), chills (0.152), diarrhea (0.124), vomiting (0.118), fever (0.096), loss of appetite (0.068), lower abdominal pain (0.040), cramping (0.037), constipation (0.036), discomfort (0.033), cyst (0.030), pain in right lower abdomen (0.029), sharp pain (0.023), pain during urination (0.022), pain in upper abdomen (0.020), pelvic pain (0.017), flank pain (0.016), vaginal discharge (0.013), abdominal discomfort (0.013), dull pain (0.012), infection (0.011)
arrhythmia	slow heart rate (0.176), racing heartbeat (0.154), fainting (0.113), fast heart rate (0.112), dizziness (0.089), lightheadedness (0.068), shortness of breath (0.045), low blood pressure (0.035), weakness (0.035), lethargy (0.032), chest pressure (0.027), fatigue (0.026), chest discomfort (0.012), chest tightness (0.012), feeling tired (0.007), dehydration (0.007)
arthritis	pain (0.406), knee pain (0.098), swelling (0.092), hip pain (0.041), shoulder pain (0.037), back pain (0.030), leg pain (0.029), neck pain (0.024), infection (0.022), tenderness (0.022), redness (0.019), joint pain (0.018), arm pain (0.017), foot pain (0.012), discomfort (0.012), weakness (0.011), depression (0.011), sadness (0.011), hand pain (0.010), acute episodes (0.008), chronic back pain (0.008), wrist pain (0.008), numbness (0.007), severe pain (0.007), diarrhea (0.007), stiffness (0.006), difficulty walking (0.006), sharp pain (0.006), cyst (0.006), swollen joint (0.006), ankle pain (0.006), elbow pain (0.005)
asthma	coughing (0.242), wheezing (0.173), shortness of breath (0.134), cough with phlegm (0.076), chest tightness (0.060), chills (0.046), acute episodes (0.044), fever (0.039), dry cough (0.032), anxiety (0.022), depression (0.021), sadness (0.021), sore throat (0.020), difficulty breathing (0.019), nasal congestion (0.014), headache (0.010), fast heart rate (0.009), phlegm (0.009), chest discomfort (0.008), chest pressure (0.008), runny nose (0.007), fatigue (0.007), back pain (0.006), fast breathing (0.005), malaise (0.005), racing heartbeat (0.005), lethargy (0.005)
atopic dermatitis	skin rash (0.232), itching (0.103), redness (0.061), anxiety (0.041), infection (0.038), dry skin (0.034), lesion (0.034), swelling (0.033), chills (0.022), acute episodes (0.021), sadness (0.018), depression (0.018), insomnia (0.018), coughing (0.016), headache (0.016)

atrial fibrillation	racing heartbeat (0.066), weakness (0.042), fast heart rate (0.035), shortness of breath (0.034), dizziness (0.028), low blood pressure (0.023), fainting (0.021), lethargy (0.018), lightheadedness (0.017), chest pressure (0.013), fatigue (0.013), confusion (0.011), mental confusion (0.010), slow heart rate (0.010), coughing (0.008), chest discomfort (0.008), bruising (0.008)
bed bug bite	skin rash (0.329), itching (0.173), anxiety (0.048), infection (0.029), sadness (0.026), depression (0.026), red spots (0.018), skin irritation (0.018), sweating (0.016), eye pain (0.015), lesion (0.012), substance abuse (0.011), hallucination (0.009), swollen feet (0.009), skin lesion (0.009), brief visual or sensory abnormality (0.009)
bell's palsy	numbness (0.308), weakness (0.198), headache (0.134), facial paralysis (0.071), ear pain (0.052), slurred speech (0.051), paralysis (0.046), facial pain (0.040), neck pain (0.038), facial swelling (0.037), tongue numbness (0.031), asymmetry (0.026), blurred vision (0.024), drooping of upper eyelid (0.020), lesion (0.019), malnutrition (0.019), difficulty swallowing (0.018), double vision (0.016)
bipolar disorder	depression (0.183), sadness (0.183), anxiety (0.138), thoughts of suicide (0.056), agitation (0.042), manic episode (0.033), crying (0.030), racing thoughts (0.030), confusion (0.026), screaming (0.026), insomnia (0.025), hearing voices (0.023), paranoia (0.023), substance abuse (0.018), falling (0.015), mental confusion (0.014), lethargy (0.014), visual hallucinations (0.012), wandering and getting lost (0.011), seizures (0.010), chronic back pain (0.010), malnutrition (0.009), slurred speech (0.009), fast heart rate (0.009), panic attack (0.009), unsteady gait (0.009), fatigue (0.008), leaking of urine (0.008), aggression (0.007), low blood pressure (0.007), sleepiness (0.006), tremor (0.006), constipation (0.006), hunger (0.006), delirium (0.005)
bladder cancer	pain (0.112), blood in urine (0.092), abdominal pain (0.039), weakness (0.033), urinary retention (0.033), fever (0.032), lethargy (0.029), flank pain (0.027), infection (0.026), fatigue (0.025), pain during urination (0.023), discomfort (0.018), bowel obstruction (0.018), low blood pressure (0.018)
bone fracture	pain (0.355), swelling (0.101), hip pain (0.054), falling (0.051), bruising (0.050), tenderness (0.047), physical deformity (0.046), back pain (0.037), ankle pain (0.027), knee pain (0.026), shoulder pain (0.025), neck pain (0.023), wrist pain (0.021), rib pain (0.021), arm pain (0.018), leg pain (0.017), numbness (0.016), foot pain (0.015), hand pain (0.014), severe pain (0.012), elbow pain (0.011), fainting (0.011), ice pack (0.008), bleeding (0.007)
bone infection	infection (0.257), pain (0.165), fever (0.083), redness (0.076), back pain (0.076), swelling (0.075), chills (0.070), foot pain (0.042), chronic back pain (0.025), depression (0.023), sadness (0.023), tenderness (0.023), weakness (0.020), confusion (0.015), hip pain (0.012), night sweats (0.011), leg pain (0.011), malaise (0.010), discomfort (0.010), incontinence (0.009), fast heart rate (0.009), watery diarrhea (0.009), low blood pressure (0.008), mental confusion (0.007), cavity (0.006)
borderline personality disorder	depression (0.343), sadness (0.342), anxiety (0.139), thoughts of suicide (0.076), abdominal pain (0.057), crying (0.054), seizures (0.037), agitation (0.023), loss of appetite (0.022), screaming (0.022), panic attack (0.015), weight loss (0.014), confusion (0.013), impulsivity (0.009), unsteady gait (0.009), mood swings (0.009), substance abuse (0.009), anger (0.009), visual hallucinations (0.008), wandering and getting lost (0.008), cramping (0.007), nightmares (0.007)
brain tumor	weakness (0.176), seizures (0.175), headache (0.123), confusion (0.117), mental confusion (0.096), lethargy (0.050), lesion (0.048), infection (0.043), vomiting (0.037), dizziness (0.034), numbness (0.033), unsteady gait (0.033), slurred speech (0.029), nausea (0.024), anxiety (0.020), memory loss (0.019), incontinence (0.019), double vision (0.017), vertigo (0.016), problems with coordination (0.016), fatigue (0.016), blurred vision (0.015), paralysis (0.013), difficulty speaking (0.011), weakness of one side of the body (0.011), disorientation (0.011), malnutrition (0.010), unsteadiness (0.010), facial pain (0.010)
breast cancer	nausea (0.066), fever (0.052), weakness (0.043), shortness of breath (0.041), chills (0.026), dizziness (0.024), diarrhea (0.023), lethargy (0.023), fatigue (0.022), infection (0.019), back pain (0.017), coughing (0.017), headache (0.017)
bronchitis	coughing (0.620), cough with phlegm (0.215), fever (0.117), chills (0.113), shortness of breath (0.097), wheezing (0.090), sore throat (0.074), dry cough (0.047), nasal congestion (0.039), phlegm (0.037), chest tightness (0.032), fatigue (0.028), lethargy (0.025), runny nose (0.022), malaise (0.019), persistent cough (0.019), chest pain worsened by breathing (0.014), chest discomfort (0.014), rib pain (0.012), headache (0.011), coughing up blood (0.011), low oxygen in the body (0.011), difficulty breathing (0.008), laryngitis (0.008), stuffy nose (0.007), anxiety (0.007)

burn	pain (0.119), redness (0.091), blister (0.063), skin rash (0.028), indigestion (0.028), infection (0.023), heartburn (0.023), depression (0.015), sadness (0.015), itching (0.013), burning sensation (0.011), swelling (0.010), discomfort (0.008), severe pain (0.008), ice pack (0.008), pain during urination (0.008), peeling skin (0.008), heat therapy (0.007), pain in upper abdomen (0.006)
cardiac arrest	seizures (0.019), fainting (0.015), low blood pressure (0.013), depression (0.011), sadness (0.011), bruising (0.011), thoughts of suicide (0.010), slow heart rate (0.010), crying (0.009), low body temperature (0.008), agitation (0.008), anxiety (0.008), respiratory distress (0.006), screaming (0.005), shivering (0.004), slurred speech (0.004), fluid in the lungs (0.004)
cardiomyopathy	shortness of breath (0.064), coughing (0.045), fast heart rate (0.040), chest pressure (0.028), racing heartbeat (0.028), low blood pressure (0.027), fainting (0.026), lightheadedness (0.021), shortness of breath while lying down (0.021), regurgitation (0.020), dizziness (0.014), weight gain (0.013), lethargy (0.013), fatigue (0.011), dry cough (0.009), anxiety (0.008), cavity (0.008), fluid in the lungs (0.007), chest discomfort (0.007), weakness (0.006), cough with phlegm (0.006)
carpal tunnel syndrome	numbness (0.175), pain (0.167), hand pain (0.094), weakness (0.083), arm pain (0.071), wrist pain (0.060), swelling (0.054), hand numbness (0.041), redness (0.030), pins and needles (0.024), shoulder pain (0.024), vertigo (0.020), hand swelling (0.016), neck pain (0.016), infection (0.014), depression (0.011), sadness (0.011), anxiety (0.011), chronic back pain (0.010), back pain (0.010), malnutrition (0.010), severe pain (0.008), unsteadiness (0.008), dry skin (0.008)
cataracts	depression (0.043), sadness (0.042), eye pain (0.035), weakness (0.031), blurred vision (0.020), vision loss (0.020), vertigo (0.018), constipation (0.017), fainting (0.017), headache (0.014), slow heart rate (0.014), dizziness (0.013), leaking of urine (0.012), difficulty swallowing (0.010)
celiac disease	pain (0.458), nausea (0.232), diarrhea (0.195), abdominal pain (0.194), vomiting (0.101), cramping (0.045), pain in upper abdomen (0.041), weight loss (0.040), dizziness (0.033), fatigue (0.032), lethargy (0.031), dehydration (0.030), chills (0.030), watery diarrhea (0.026), constipation (0.024), weakness (0.024), infection (0.022), lightheadedness (0.020), back pain (0.020), loss of appetite (0.019), sharp pain (0.018), tenderness (0.014), yellow skin and eyes (0.013), malnutrition (0.012), upper abdominal pain (0.012), flushing (0.012), blood in stool (0.012), lower abdominal pain (0.012), abdominal discomfort (0.012)
cellulitis	redness (0.508), swelling (0.404), pain (0.172), infection (0.124), fever (0.074), chills (0.074), skin rash (0.042), tenderness (0.039), leg pain (0.027), foot pain (0.022), itching (0.020), blister (0.016), lesion (0.014), pimple (0.013), hand swelling (0.012), facial swelling (0.011), scab (0.009), lower extremity pain (0.008)
cervical cancer	pain (0.329), abdominal pain (0.175), nausea (0.129), vomiting (0.078), bleeding (0.068), vaginal bleeding (0.064), chills (0.052), diarrhea (0.046), bleeding from anus (0.045), lethargy (0.044), anxiety (0.042), low blood pressure (0.039), discomfort (0.033), sadness (0.033), depression (0.032), redness (0.028), fatigue (0.027), pelvic pain (0.021), abdominal distension (0.019)
cervical radiculopathy	pain (0.557), numbness (0.379), neck pain (0.313), shoulder pain (0.193), weakness (0.177), arm pain (0.157), back pain (0.084), pins and needles (0.047), tenderness (0.032), hand numbness (0.026), discomfort (0.020), severe pain (0.020), radiating pain (0.019), sharp pain (0.017), depression (0.017), sadness (0.016), anxiety (0.016), chronic back pain (0.015)
chicken pox	skin rash (0.470), itching (0.105), fever (0.087), burning sensation (0.042), headache (0.034), infection (0.034), lesion (0.032), chills (0.031), pain in upper abdomen (0.027), red rashes (0.027), sore throat (0.027), numbness (0.026), malnutrition (0.026), redness (0.025), nerve pain (0.025), stinging sensation (0.018), neck swelling (0.018), ear pain (0.015), ache (0.014), nausea (0.013), night sweats (0.012), pins and needles (0.011)
chlamydia	pain (0.385), pain during urination (0.196), nausea (0.152), vaginal discharge (0.148), tenderness (0.130), abdominal pain (0.099), pelvic pain (0.089), cramping (0.078), chills (0.073), infection (0.065), vaginal bleeding (0.057), bleeding (0.054), pain in lower abdomen (0.052), discomfort (0.050), lower abdominal pain (0.049), vomiting (0.038), fever (0.038), discharge from penis (0.034), cyst (0.033), itching (0.032)
cholangiocarcinoma	fever (0.268), pain (0.201), nausea (0.190), abdominal pain (0.189), yellow skin and eyes (0.112), vomiting (0.099), chills (0.097), weakness (0.064), lethargy (0.057), fluid in the abdomen (0.056), pain in upper abdomen (0.053), dark urine (0.037), shortness of breath (0.030), constipation (0.029), low blood pressure (0.026), infection (0.026), abdominal distension (0.025), fatigue (0.022), discomfort (0.021), confusion (0.017)

cholecystitis	pain (0.621), abdominal pain (0.245), nausea (0.225), vomiting (0.123), tenderness (0.061), fever (0.057), chills (0.056), pain in upper abdomen (0.052), diarrhea (0.051), pain in upper-right abdomen (0.026), abdominal cramping from gallstones (0.023), yellow skin and eyes (0.023), constipation (0.017), discomfort (0.015), infection (0.014), upper abdominal pain (0.014), sharp pain (0.013), lethargy (0.013), loss of appetite (0.011), pain during urination (0.009)
chronic kidney disease	kidney failure (0.041), low blood pressure (0.033), infection (0.030), weakness (0.029), fever (0.022), diarrhea (0.020), coughing (0.020), shortness of breath (0.019), vomiting (0.019), lethargy (0.015), chills (0.012), bleeding (0.011), abdominal pain (0.010), fatigue (0.009), low oxygen in the body (0.008), cough with phlegm (0.007), malaise (0.007), confusion (0.006), fluid in the lungs (0.006), redness (0.005), mental confusion (0.005)
chronic obstructive pulmonary disease	coughing (0.186), shortness of breath (0.112), cough with phlegm (0.071), wheezing (0.066), low oxygen in the body (0.026), acute episodes (0.020), lethargy (0.015), fever (0.013), chills (0.013), chest tightness (0.012), respiratory distress (0.012), difficulty breathing (0.011), weakness (0.010), phlegm (0.010), fatigue (0.008), fast breathing (0.008), chronic cough (0.006), anxiety (0.006), low blood pressure (0.006), dry cough (0.006), fast heart rate (0.006), sadness (0.005), depression (0.005), confusion (0.005), infection (0.005), chest pressure (0.005)
chronic pain	pain (0.556), back pain (0.114), abdominal pain (0.074), sadness (0.070), depression (0.070), chronic back pain (0.058), nausea (0.053), anxiety (0.041), leg pain (0.038), weakness (0.031), numbness (0.026), neck pain (0.025), vomiting (0.023), knee pain (0.022), diarrhea (0.021), shoulder pain (0.020), hip pain (0.019), constipation (0.019), tenderness (0.018), severe pain (0.018), crying (0.017), falling (0.016)
cirrhosis of the liver	fluid in the abdomen (0.229), abdominal pain (0.097), pain (0.096), nausea (0.072), yellow skin and eyes (0.071), swollen veins in the lower esophagus (0.067), confusion (0.055), mental confusion (0.049), lethargy (0.045), vomiting (0.044), weakness (0.039), diarrhea (0.037), abdominal distension (0.030), vomiting blood (0.030), chills (0.029), fatigue (0.028), bleeding (0.027), dark stool from digested blood (0.025), flapping tremor (0.025), shortness of breath (0.022), fever (0.020), low blood pressure (0.018), weight gain (0.016), discomfort (0.015), bleeding from anus (0.014), swelling (0.013), infection (0.013), lightheadedness (0.008), abdominal discomfort (0.007), bruising (0.007), kidney failure (0.007), coughing (0.007), lesion (0.007), dizziness (0.006), pain in upper abdomen (0.006)
colon cancer	nausea (0.179), abdominal pain (0.137), vomiting (0.107), pain (0.104), diarrhea (0.076), constipation (0.060), bowel obstruction (0.047), weakness (0.043), lethargy (0.038), fatigue (0.030), chills (0.029), dehydration (0.028), shortness of breath (0.027), discomfort (0.025), low blood pressure (0.022), dizziness (0.022), fast heart rate (0.020), infection (0.019), blood in stool (0.017), loss of appetite (0.016), cramping (0.015), back pain (0.012), failure to thrive (0.012), poor appetite (0.011), sadness (0.010), dark stool from digested blood (0.010), depression (0.010), weight loss (0.010)
common cold	chills (0.083), sore throat (0.075), coughing (0.071), fever (0.057), nasal congestion (0.039), headache (0.036), runny nose (0.029), numbness (0.024), dry cough (0.019), nausea (0.018), fatigue (0.014), ear pain (0.013), dizziness (0.012), diarrhea (0.011), malaise (0.011), fainting (0.010), lethargy (0.009), lightheadedness (0.008), sneezing (0.008), discomfort (0.008), shivering (0.007), stuffy nose (0.007), racing heartbeat (0.006), sinus pressure (0.006), feeling cold (0.005), foot pain (0.004), pins and needles (0.004), sweating (0.004), phlegm (0.004), skin rash (0.004), sensitivity to light (0.004), night sweats (0.004), low body temperature (0.004), mild cough (0.003), eye redness (0.003), cough with phlegm (0.003), loss of appetite (0.003), head congestion (0.003), leg pain (0.003), ache (0.003)
concussion	headache (0.240), nausea (0.176), neck pain (0.118), dizziness (0.112), tenderness (0.082), blurred vision (0.046), confusion (0.045), fainting (0.042), mental confusion (0.040), bruising (0.038), falling (0.038), sensitivity to light (0.031), vomiting (0.027), lightheadedness (0.021), amnesia (0.019), shoulder pain (0.018), back pain (0.017), numbness (0.016), difficulty concentrating (0.015), memory loss (0.014), double vision (0.014), lethargy (0.011), vertigo (0.011), elbow pain (0.010), stiffness (0.010), rib pain (0.009), unsteady gait (0.009), fatigue (0.008), persistent headache (0.007), knee pain (0.007), ache (0.007), arm pain (0.007), rapid involuntary eye movement (0.006), disorientation (0.006), ice pack (0.006), severe headache (0.005), malnutrition (0.005), fear of loud sounds (0.004), facial pain (0.004)

congestive heart failure	shortness of breath (0.293), swelling (0.097), coughing (0.095), shortness of breath while lying down (0.080), weight gain (0.065), fluid in the lungs (0.052), wheezing (0.040), low oxygen in the body (0.034), cough with phlegm (0.027), lethargy (0.024), difficulty breathing (0.021), chest pressure (0.018), dry cough (0.017), fatigue (0.017), respiratory distress (0.017), chest tightness (0.013), fast breathing (0.013), swelling in extremities (0.012), kidney failure (0.011), regurgitation (0.009), weakness (0.008), phlegm (0.008), low blood pressure (0.008), chest discomfort (0.006), slow heart rate (0.004), mild cough (0.004), chronic cough (0.004)
coronary artery disease	shortness of breath (0.051), regurgitation (0.022), fainting (0.022), low blood pressure (0.021), lesion (0.021), chest pressure (0.018), depression (0.016), sadness (0.015), weakness (0.013), back pain (0.012), malnutrition (0.011), discomfort (0.010), chest discomfort (0.008), leg pain (0.007), infection (0.007), leg pain during exercise (0.006)
crohn's disease	pain (0.548), abdominal pain (0.292), nausea (0.276), acute episodes (0.245), diarrhea (0.201), vomiting (0.133), chills (0.093), fever (0.061), bowel obstruction (0.043), cramping (0.042), tenderness (0.038), dehydration (0.027), discomfort (0.025), lower abdominal pain (0.022), blood in stool (0.020), infection (0.019), rectal pain (0.016), bleeding from anus (0.015), constipation (0.013), lethargy (0.013), fatigue (0.012), pain in upper abdomen (0.011), low blood pressure (0.010), pain during urination (0.009), watery diarrhea (0.009), abdominal discomfort (0.009), weight loss (0.008), belching (0.008), abdominal tenderness (0.008), abdominal distension (0.007), lightheadedness (0.007), sharp pain (0.006), bone loss (0.005)
deep vein thrombosis	pain (0.284), swelling (0.237), leg pain (0.095), calf pain (0.052), shortness of breath (0.037), lower extremity pain (0.032), coughing (0.028), fast heart rate (0.024), malnutrition (0.020), weakness (0.020), tenderness (0.019), bleeding (0.019), coughing up blood (0.017), discomfort (0.014), arm pain (0.013), redness (0.011)
dementia	confusion (0.121), mental confusion (0.093), fainting (0.042), agitation (0.038), hip pain (0.026), lethargy (0.020), weakness (0.018), delirium (0.018), low blood pressure (0.017), seizures (0.011), bruising (0.011), wandering and getting lost (0.011), low oxygen in the body (0.011), depression (0.011), sadness (0.010), incontinence (0.010), failure to thrive (0.009), infection (0.009), falling (0.009), leaking of urine (0.008), dehydration (0.007), paranoia (0.007), memory loss (0.007), screaming (0.006), visual hallucinations (0.005), confusion in the evening hours (0.005), unsteady gait (0.004), aggression (0.004), fast breathing (0.004), difficulty swallowing (0.003), malnutrition (0.003), slow heart rate (0.003), kidney failure (0.003)
diverticulitis	pain (0.657), abdominal pain (0.271), nausea (0.224), diarrhea (0.152), chills (0.121), fever (0.072), vomiting (0.059), constipation (0.057), tenderness (0.051), cramping (0.044), lower abdominal pain (0.041), acute episodes (0.026), discomfort (0.026), pain during urination (0.021), loss of appetite (0.017), flank pain (0.015), blood in stool (0.014), bowel obstruction (0.014), abdominal discomfort (0.012), bleeding from anus (0.012), watery diarrhea (0.010), blood in urine (0.010), pain in upper abdomen (0.010), pain in lower abdomen (0.009), severe pain (0.007), abdominal tenderness (0.006)
diverticulosis	pain (0.187), diarrhea (0.116), abdominal pain (0.110), nausea (0.084), bleeding from anus (0.084), cramping (0.060), bleeding (0.059), constipation (0.040), vomiting (0.038), lightheadedness (0.034), dizziness (0.026), lower abdominal pain (0.026), discomfort (0.025), blood in stool (0.020), dark stool from digested blood (0.020), chills (0.018), lesion (0.017), cyst (0.017)
drug withdrawal	sadness (0.186), depression (0.184), anxiety (0.182), nausea (0.083), seizures (0.049), thoughts of suicide (0.049), substance abuse (0.038), diarrhea (0.037), vomiting (0.035), panic attack (0.030), insomnia (0.027), crying (0.024), agitation (0.024), racing heartbeat (0.021), chills (0.021), fast heart rate (0.021), confusion (0.020), severe anxiety (0.014), cramping (0.014), racing thoughts (0.013), mental confusion (0.012), restlessness (0.012), visual hallucinations (0.011), hopelessness (0.011), paranoia (0.011), hallucination (0.010), muscle pain (0.009), tremor (0.008), sleepiness (0.007), hearing voices (0.007), screaming (0.006), leg pain (0.006), discomfort in upper abdomen (0.006), guilt (0.006), chronic back pain (0.006), bruising (0.006), falling (0.006), abdominal pain (0.006), nightmares (0.005), runny nose (0.005), joint pain (0.005), loss of interest (0.004), impulsivity (0.004), lethargy (0.004), slowness in activity and thought (0.004), restless legs syndrome (0.004), trembling (0.004), wheezing (0.004), dry skin (0.004), wandering and getting lost (0.004), fear (0.004), nasal congestion (0.003), weight loss (0.003), blotchy rash (0.003)

ectopic pregnancy	pain (0.537), bleeding (0.204), vaginal bleeding (0.181), abdominal pain (0.167), cramping (0.155), spotting (0.154), nausea (0.104), cyst (0.067), tenderness (0.055), lower abdominal pain (0.048), pelvic pain (0.040), diarrhea (0.031), vaginal discharge (0.023), discomfort (0.020), vomiting (0.016), back pain (0.015), vaginal pain (0.014), lightheadedness (0.011)
encephalitis	confusion (0.160), mental confusion (0.117), headache (0.112), fever (0.104), seizures (0.080), lethargy (0.052), dizziness (0.047), weakness (0.035), fatigue (0.031), stiff neck (0.026), slurred speech (0.024), blurred vision (0.022), severe headache (0.021), skin rash (0.020), infection (0.019), poor appetite (0.019), delirium (0.019), agitation (0.017), neck pain (0.017), fluid in the abdomen (0.016), anxiety (0.015), blood in urine (0.015), low blood pressure (0.015), severe pain (0.014), visual hallucinations (0.013), forgetfulness (0.013), high fever (0.012), lesion (0.012), blister (0.012)
encephalopathy	confusion (0.350), mental confusion (0.257), fluid in the abdomen (0.159), flapping tremor (0.117), lethargy (0.075), yellow skin and eyes (0.042), infection (0.040), seizures (0.038), swollen veins in the lower esophagus (0.033), depression (0.025), sadness (0.025), unsteady gait (0.021), agitation (0.021), fatigue (0.016), tremor (0.015), weakness (0.015), slurred speech (0.014), delirium (0.014), abdominal distension (0.011), visual hallucinations (0.011), falling (0.009), kidney failure (0.009), incontinence (0.008)
endometrial cancer	nausea (0.096), fever (0.069), vaginal bleeding (0.042), vomiting (0.040), bowel obstruction (0.033), fatigue (0.029), weight loss (0.028), pain in upper abdomen (0.025), lethargy (0.024), constipation (0.023), diarrhea (0.022), abdominal pain (0.020), regurgitation (0.018), abdominal distension (0.018), weakness (0.018), bleeding (0.017)
endometriosis	pain (0.567), nausea (0.287), abdominal pain (0.202), pelvic pain (0.142), vomiting (0.113), diarrhea (0.077), cramping (0.076), tenderness (0.070), bleeding (0.057), vaginal bleeding (0.053), constipation (0.047), lower abdominal pain (0.046), cyst (0.037), chills (0.034), spotting (0.026), vaginal discharge (0.021), flank pain (0.019), severe pain (0.017), pain in lower abdomen (0.016), depression (0.016), sadness (0.016), acute episodes (0.014), loss of appetite (0.014), vaginal pain (0.012), pain during urination (0.012), discomfort (0.012), lethargy (0.011), bleeding from anus (0.010), fatigue (0.010)
epididymitis	testicle pain (0.577), pain (0.567), swelling (0.283), tenderness (0.156), groin pain (0.126), infection (0.098), pain during urination (0.082), chills (0.062), blood in urine (0.055), nausea (0.053), redness (0.052), discomfort (0.040), lump (0.040), lower abdominal pain (0.030), fever (0.027), cyst (0.023), severe pain (0.019), vomiting (0.018), flank pain (0.015), drooping of upper eyelid (0.014), rectal pain (0.013), pelvic pain (0.011), frequent urge to urinate (0.011)
epilepsy	seizures (0.604), confusion (0.059), headache (0.053), depression (0.048), sadness (0.048), brief visual or sensory abnormality (0.046), mental confusion (0.044), weakness (0.040), anxiety (0.028), falling (0.018), incontinence (0.017), lethargy (0.016), dizziness (0.014), fainting (0.013), weakness of one side of the body (0.012), leaking of urine (0.012), agitation (0.012), unsteady gait (0.011), tremor (0.008), infection (0.008), fast heart rate (0.007), tenderness (0.007), problems with coordination (0.007), low blood pressure (0.006), sleepiness (0.006)
esophageal cancer	nausea (0.138), weakness (0.103), vomiting (0.099), difficulty swallowing (0.072), abdominal pain (0.065), lethargy (0.065), fatigue (0.063), dehydration (0.060), coughing (0.049), fast heart rate (0.048), shortness of breath (0.045), low blood pressure (0.043), failure to thrive (0.024), vomiting blood (0.023), weight loss (0.021), lightheadedness (0.018), diarrhea (0.017), phlegm (0.015), constipation (0.014), fever (0.013), back pain (0.012), rib pain (0.012), belching (0.012), malaise (0.011), redness (0.011), dark urine (0.010), yellow skin and eyes (0.008), gagging (0.008)
fibroids	pain (0.423), bleeding (0.201), vaginal bleeding (0.181), abdominal pain (0.136), nausea (0.125), cramping (0.099), pelvic pain (0.073), tenderness (0.073), heavy or prolonged periods (0.053), cyst (0.053), spotting (0.051), lower abdominal pain (0.047), chills (0.045), pain during urination (0.034), lightheadedness (0.033), dizziness (0.031), constipation (0.030), fever (0.029), discomfort (0.028), vaginal discharge (0.026)

fibromyalgia	pain (0.381), depression (0.112), sadness (0.112), nausea (0.106), back pain (0.068), anxiety (0.068), weakness (0.045), chills (0.035), abdominal pain (0.033), headache (0.025), numbness (0.025), lethargy (0.025), chronic back pain (0.022), fatigue (0.020), diarrhea (0.020), acute episodes (0.019), tenderness (0.016), arm pain (0.016), infection (0.014), constipation (0.013), neck pain (0.012), flank pain (0.011), incontinence (0.011), discomfort (0.011), shoulder pain (0.010), leg pain (0.010), leaking of urine (0.010), knee pain (0.010), hip pain (0.009), fever (0.009), chest tightness (0.009), dizziness (0.008), muscle pain (0.008), crying (0.008), vertigo (0.007), severe pain (0.007), dehydration (0.007), bruising (0.006), pelvic pain (0.006), sensitivity to light (0.006), chest pressure (0.005), lesion (0.005)
gallstones	pain (0.663), nausea (0.281), abdominal pain (0.227), pain in upper abdomen (0.117), vomiting (0.113), chills (0.057), tenderness (0.043), abdominal cramping from gallstones (0.031), yellow skin and eyes (0.025), pain in upper-right abdomen (0.025), diarrhea (0.025), fever (0.023), flank pain (0.020), upper abdominal pain (0.020), constipation (0.015), discomfort (0.014), dark urine (0.011), loss of appetite (0.010), severe pain (0.009), belching (0.007), discomfort in upper abdomen (0.007), intermittent pain (0.006), cyst (0.006)
gastritis	pain (0.420), nausea (0.284), vomiting (0.198), abdominal pain (0.184), pain in upper abdomen (0.162), diarrhea (0.080), chills (0.048), vomiting blood (0.029), tenderness (0.028), discomfort (0.027), constipation (0.022), cramping (0.021), anxiety (0.019), discomfort in upper abdomen (0.018), depression (0.016), sadness (0.016), dizziness (0.014), dark stool from digested blood (0.013), weight loss (0.012), upper abdominal pain (0.012)
gastroparesis	pain (0.517), vomiting (0.516), nausea (0.468), abdominal pain (0.292), acute episodes (0.049), pain in upper abdomen (0.048), diarrhea (0.039), gagging (0.022), depression (0.020), sadness (0.019), dehydration (0.019), constipation (0.015), fast heart rate (0.015), anxiety (0.015), tenderness (0.014), cramping (0.012), upper abdominal pain (0.012), chills (0.009), discomfort (0.008), severe anxiety (0.007), crying (0.007), abdominal tenderness (0.007), loss of appetite (0.007), flank pain (0.006)
glaucoma	eye pain (0.082), vision loss (0.060), headache (0.036), redness (0.031), blurred vision (0.030), eye redness (0.021), depression (0.018), sadness (0.018), infection (0.016), weakness (0.015), fainting (0.014), bone loss (0.014)
goiter	weakness (0.055), racing heartbeat (0.055), lump (0.045), neck pain (0.044), shortness of breath (0.042), cyst (0.038), dizziness (0.031), difficulty swallowing (0.031), sore throat (0.030), ear pain (0.028), chills (0.028), memory loss (0.023), chest pressure (0.022), fast heart rate (0.022), neck swelling (0.020), indigestion (0.019)
gout	pain (0.274), swelling (0.246), redness (0.103), acute episodes (0.097), foot pain (0.092), knee pain (0.073), ankle pain (0.053), toe pain (0.045), wrist pain (0.022), infection (0.021), elbow pain (0.017), tenderness (0.017), leg pain (0.017), hand pain (0.016), joint pain (0.014), fever (0.011), hand swelling (0.010), difficulty walking (0.009), hip pain (0.008), kidney failure (0.008), limping (0.007), chills (0.007), arm pain (0.006), lump on the skin or joint (0.006), swollen knee (0.005)
groin hernia	pain (0.502), groin pain (0.144), abdominal pain (0.075), swelling (0.054), nausea (0.052), constipation (0.043), vomiting (0.040), lump (0.040), testicle pain (0.036), discomfort (0.035), bowel obstruction (0.021), sharp pain (0.019), tenderness (0.016), urinary retention (0.016), diarrhea (0.015), lower abdominal pain (0.013), chills (0.013)
heart attack	pain (0.177), nausea (0.104), anxiety (0.052), chest pressure (0.047), shortness of breath (0.041), racing heartbeat (0.035), shoulder pain (0.032), chest discomfort (0.030), numbness (0.028), discomfort (0.024), dizziness (0.022), arm pain (0.020), depression (0.020), sadness (0.020), pain in upper abdomen (0.018), neck pain (0.017), chest tightness (0.014), jaw pain (0.014), vomiting (0.013), discomfort in upper abdomen (0.012), indigestion (0.010), low blood pressure (0.010), lesion (0.009), weight gain (0.009), agitation (0.008), fluid in the lungs (0.008), malnutrition (0.008), fainting (0.007), arm discomfort (0.006), weakness (0.006), slow heart rate (0.006), lightheadedness (0.006), drooping of upper eyelid (0.006), stuffy nose (0.006), difficulty breathing (0.005), diarrhea (0.005)
hepatitis a	pain (0.216), nausea (0.167), abdominal pain (0.124), vomiting (0.112), diarrhea (0.100), yellow skin and eyes (0.089), fever (0.087), chills (0.061), dark urine (0.047), tenderness (0.034), lethargy (0.030), fatigue (0.029), pain in upper abdomen (0.026), loss of appetite (0.024), depression (0.021), sadness (0.021), fluid in the abdomen (0.021), coughing (0.019), skin rash (0.018),

	weakness (0.017), night sweats (0.014), infection (0.014), itching (0.012)
hepatitis b	abdominal pain (0.051), chills (0.035), lethargy (0.032), back pain (0.027), nausea (0.027), fatigue (0.027), weakness (0.024), yellow skin and eyes (0.023), dizziness (0.020), fluid in the abdomen (0.019), depression (0.017), sadness (0.017), discomfort (0.014), infection (0.012), constipation (0.011), cough with phlegm (0.011), lesion (0.010), coughing (0.010), diarrhea (0.010), fever (0.010), vomiting blood (0.010), chronic back pain (0.008)
hepatitis c	pain (0.112), depression (0.063), sadness (0.063), abdominal pain (0.047), nausea (0.037), chills (0.033), diarrhea (0.025), anxiety (0.024), infection (0.022), fever (0.021), vomiting (0.020), confusion (0.018), substance abuse (0.017), fluid in the abdomen (0.015), lethargy (0.014), thoughts of suicide (0.013), coughing (0.013), back pain (0.010), fatigue (0.010), yellow skin and eyes (0.010), mental confusion (0.009), chronic back pain (0.009)
hiatal hernia	pain (0.157), nausea (0.144), vomiting (0.095), abdominal pain (0.076), depression (0.037), sadness (0.037), constipation (0.035), diarrhea (0.032), pain in upper abdomen (0.028), shortness of breath (0.027), chills (0.026), discomfort (0.024), weakness (0.021), chest pressure (0.018), anxiety (0.017), tenderness (0.016), fainting (0.015), weakness of one side of the body (0.015), back pain (0.015)
high blood pressure	headache (0.052), sadness (0.041), depression (0.041), dizziness (0.032), shortness of breath (0.029), anxiety (0.021), lightheadedness (0.016), fainting (0.014), regurgitation (0.012), weakness (0.011)
high cholesterol	nausea (0.027), sadness (0.027), weakness (0.027), depression (0.027), dizziness (0.026), fainting (0.019), discomfort (0.018), anxiety (0.018), shortness of breath (0.014), lethargy (0.013)
hiv/aids	coughing (0.080), depression (0.059), sadness (0.059), fever (0.056), cough with phlegm (0.044), chills (0.038), confusion (0.035), infection (0.034), oral thrush (0.033), vomiting (0.032), mental confusion (0.031), shortness of breath (0.029), nausea (0.029), lethargy (0.028), weakness (0.027), lesion (0.023), skin rash (0.022), low blood pressure (0.017), dry cough (0.016), fatigue (0.015), diarrhea (0.015), constipation (0.015), leg pain (0.014), difficulty swallowing (0.013), malaise (0.012), abdominal pain (0.011), watery diarrhea (0.011), seizures (0.010), falling (0.009), anxiety (0.009), fast breathing (0.009), kidney failure (0.008)
hives	skin rash (0.334), itching (0.222), swelling (0.166), redness (0.098), facial swelling (0.043), tongue swelling (0.024), shortness of breath (0.020), difficulty swallowing (0.019), wheezing (0.019), raised area of skin (0.016), swelling under the skin (0.016), red rashes (0.016), difficulty breathing (0.015), chest tightness (0.012), sore throat (0.012), hand swelling (0.010), lump (0.008)
hyperthyroidism	racing heartbeat (0.117), fast heart rate (0.075), dizziness (0.070), weight loss (0.055), anxiety (0.046), depression (0.036), sadness (0.036), vomiting (0.031), nausea (0.030), fainting (0.029), shortness of breath (0.024), lethargy (0.023), abdominal pain (0.023), fatigue (0.021), weakness (0.021), sharp pain (0.019), loss of appetite (0.018), low blood pressure (0.018), swelling (0.018), chest tightness (0.017), vertigo (0.016), dehydration (0.015), wheezing (0.014), constipation (0.013), panic attack (0.013), bruising (0.012), headache (0.010), lightheadedness (0.010), infection (0.010)
hypoglycemia	confusion (0.073), fainting (0.053), seizures (0.050), weakness (0.039), mental confusion (0.027), slurred speech (0.022), low blood pressure (0.022), low body temperature (0.019), depression (0.015), sadness (0.015), loss of appetite (0.012), dizziness (0.011), lethargy (0.011), kidney failure (0.009), lightheadedness (0.009), sweating (0.008), unsteady gait (0.007), slow heart rate (0.006), snoring (0.006), dehydration (0.006), weight loss (0.006), anxiety (0.005), sleepiness (0.005), diarrhea (0.004), coma (0.004), poor appetite (0.004), blurred vision (0.004), low oxygen in the body (0.003), feeling cold (0.003), malnutrition (0.003), wandering and getting lost (0.003), thirst (0.003), drooling (0.003)
hyponatremia	weakness (0.107), lethargy (0.064), nausea (0.062), confusion (0.053), dizziness (0.045), mental confusion (0.044), fatigue (0.039), diarrhea (0.038), vomiting (0.036), dehydration (0.034), seizures (0.029), coughing (0.023), low blood pressure (0.019), lightheadedness (0.017), constipation (0.017), anxiety (0.015), loss of appetite (0.014), weight loss (0.014), malaise (0.013), chills (0.012)

hypothyroidism	depression (0.040), sadness (0.040), nausea (0.036), weakness (0.026), anxiety (0.021), dizziness (0.021), lethargy (0.019), fainting (0.017), diarrhea (0.015), fatigue (0.014), vomiting (0.012), confusion (0.010), mental confusion (0.009), lightheadedness (0.009), malaise (0.009), racing heartbeat (0.009), constipation (0.008), seizures (0.007), chills (0.006), vertigo (0.005), discomfort (0.005), headache (0.005), dehydration (0.005), chest pressure (0.005), shortness of breath (0.005), numbness (0.004), leg pain (0.004), low blood pressure (0.004), bone loss (0.003), weight loss (0.003), slow heart rate (0.003), cough with phlegm (0.003), drooping of upper eyelid (0.003)
insect bites and stings	skin rash (0.193), redness (0.181), swelling (0.119), itching (0.103), lesion (0.030), fever (0.018), chills (0.018), malaise (0.014), night sweats (0.014), sore throat (0.011), red spots (0.010), bruising (0.010), stuffy nose (0.010), red rashes (0.010), fear (0.010)
iron deficiency anemia	iron deficiency (0.280), depression (0.084), sadness (0.084), bleeding (0.053), lethargy (0.049), fatigue (0.043), dizziness (0.039), weakness (0.034), lightheadedness (0.029), anxiety (0.024), malnutrition (0.022), difficulty swallowing (0.022), constipation (0.021), weight loss (0.020), restless legs syndrome (0.018), low blood pressure (0.016), nausea (0.015), headache (0.015), heavy or prolonged periods (0.013), leg pain during exercise (0.011)
irritable bowel syndrome	pain (0.283), nausea (0.253), diarrhea (0.250), abdominal pain (0.200), anxiety (0.088), cramping (0.074), sadness (0.073), vomiting (0.072), constipation (0.072), depression (0.072), discomfort (0.059), lower abdominal pain (0.033), acute episodes (0.032), drooping of upper eyelid (0.029), bone loss (0.027), loss of appetite (0.024), rectal pain (0.023), infection (0.023), seizures (0.023), dehydration (0.021), dizziness (0.020), crying (0.019), vertigo (0.019), abdominal distension (0.018), belching (0.018), weight loss (0.015), severe pain (0.015), tenderness (0.015), insomnia (0.014)
kidney infection	flank pain (0.413), pain (0.383), chills (0.287), nausea (0.257), fever (0.217), pain during urination (0.186), back pain (0.173), tenderness (0.149), vomiting (0.132), abdominal pain (0.058), blood in urine (0.057), infection (0.049), pain in lower abdomen (0.034), discomfort (0.034), diarrhea (0.023), fast heart rate (0.014), frequent urge to urinate (0.014), foul smelling urine (0.013), dehydration (0.013), fatigue (0.011), lower abdominal pain (0.011), malaise (0.011), headache (0.010), abdominal tenderness (0.009), abdominal discomfort (0.009), vaginal discharge (0.009), muscle pain (0.009), cramping (0.007), lethargy (0.007), leaking of urine (0.006), dark urine (0.006)
kidney stone	pain (0.608), flank pain (0.495), nausea (0.232), blood in urine (0.141), pain during urination (0.084), vomiting (0.083), chills (0.067), abdominal pain (0.065), back pain (0.050), tenderness (0.040), discomfort (0.019), groin pain (0.018), severe pain (0.013), fever (0.012), testicle pain (0.011), frequent urge to urinate (0.011), lower abdominal pain (0.011), dark urine (0.011), urinary retention (0.011), sharp pain (0.010), cyst (0.010), pain in lower abdomen (0.010), diarrhea (0.009), constipation (0.008), infection (0.007), pelvic pain (0.007), side pain (0.004), dull pain (0.004)
liver cancer	pain (0.259), nausea (0.128), abdominal pain (0.125), vomiting (0.092), fluid in the abdomen (0.077), weakness (0.076), yellow skin and eyes (0.064), confusion (0.057), lethargy (0.056), shortness of breath (0.047), mental confusion (0.045), diarrhea (0.042), fatigue (0.036), fever (0.035), vomiting blood (0.034), tenderness (0.034), pain in upper-right abdomen (0.028), dehydration (0.028), flank pain (0.027), fast heart rate (0.024)
low back pain	pain (0.463), back pain (0.411), numbness (0.075), tenderness (0.056), chronic back pain (0.055), weakness (0.053), neck pain (0.028), leg pain (0.025), incontinence (0.024), leaking of urine (0.023), flank pain (0.019), hip pain (0.018), discomfort (0.016), pain during urination (0.014), falling (0.013), sharp pain (0.012), severe pain (0.011), difficulty walking (0.010)
lung cancer	shortness of breath (0.129), coughing (0.108), weakness (0.086), lesion (0.046), lethargy (0.037), back pain (0.033), fever (0.032), fatigue (0.026), cough with phlegm (0.023), coughing up blood (0.022), nausea (0.022), infection (0.021), constipation (0.020), confusion (0.018), low oxygen in the body (0.017), malaise (0.016), lump (0.016), chronic cough (0.015), wheezing (0.015), fast heart rate (0.014), mental confusion (0.014), sharp pain (0.011), weight loss (0.010), dehydration (0.010), difficulty swallowing (0.010), dry cough (0.010), skin rash (0.009), chills (0.009)
lupus	pain (0.173), nausea (0.053), acute episodes (0.049), swelling (0.048), fever (0.037), diarrhea (0.034), abdominal pain (0.031), weakness (0.030), shortness of breath (0.030), chills (0.029), headache (0.024), joint pain (0.022), coughing (0.021), skin rash (0.021), seizures (0.020), fatigue (0.018), tenderness (0.018), cramping (0.015), lethargy (0.015), vomiting (0.014), infection (0.013), racing heartbeat (0.012), fast heart rate (0.009), dry cough (0.009), dizziness (0.009), cough with

	phlegm (0.008), flank pain (0.008), face rash (0.006)
lyme disease	headache (0.163), skin rash (0.163), fever (0.131), lethargy (0.124), weakness (0.112), chills (0.108), numbness (0.102), fatigue (0.100), nausea (0.090), pain (0.076), joint pain (0.067), neck pain (0.062), stiffness (0.056), infection (0.054), blurred vision (0.038), malaise (0.037), dizziness (0.035), redness (0.032), discomfort (0.029), night sweats (0.026)
major depression	depression (0.416), sadness (0.416), anxiety (0.222), thoughts of suicide (0.101), agitation (0.049), insomnia (0.035), weight loss (0.032), inability to feel pleasure (0.031), seizures (0.026), fast heart rate (0.026), poor appetite (0.024), hopelessness (0.024), loss of appetite (0.022), visual hallucinations (0.020), discomfort (0.020), tremor (0.019), racing thoughts (0.019), chronic back pain (0.016), loss of interest (0.016), hearing voices (0.016), crying (0.014), lightheadedness (0.013), constipation (0.012), delirium (0.012), substance abuse (0.011), pain during urination (0.009), falling (0.008), pain when coughing (0.008), guilt (0.008), pain worse at rest (0.008), difficulty concentrating (0.008), headache (0.008)
melanoma	weakness (0.113), confusion (0.068), mental confusion (0.055), lesion (0.053), nausea (0.045), lethargy (0.044), vomiting (0.034), fatigue (0.030), seizures (0.029), back pain (0.024), shortness of breath (0.018), numbness (0.016), dizziness (0.014)
meningitis	headache (0.450), fever (0.340), neck pain (0.205), nausea (0.157), stiffness (0.155), sensitivity to light (0.142), chills (0.134), stiff neck (0.063), back pain (0.057), vomiting (0.055), lethargy (0.046), infection (0.045), skin rash (0.043), confusion (0.041), weakness (0.037), coughing (0.035), severe headache (0.034), pain (0.034), seizures (0.033), sore throat (0.030), fatigue (0.029), malaise (0.023), dizziness (0.017), mental confusion (0.017), fear of loud sounds (0.014), diarrhea (0.013), night sweats (0.011), dry cough (0.011), ache (0.010), muscle pain (0.010)
middle ear infection	ear pain (0.694), inflammation of ear (0.158), sore throat (0.118), coughing (0.096), fever (0.069), nasal congestion (0.055), pain (0.053), ache (0.050), chills (0.049), headache (0.035), infection (0.029), redness (0.029), dizziness (0.020), leaked fluid out of blood vessels or an organ (0.017), cough with phlegm (0.015), runny nose (0.015), sinus pressure (0.014), sinus pain (0.014), ringing in the ears (0.008), discomfort (0.008)
migraine	headache (0.384), nausea (0.316), sensitivity to light (0.223), pain (0.217), vomiting (0.110), numbness (0.067), dizziness (0.062), fear of loud sounds (0.038), anxiety (0.037), neck pain (0.037), weakness (0.037), blurred vision (0.033), brief visual or sensory abnormality (0.028), depression (0.026), sadness (0.026), vertigo (0.021), chills (0.019), severe headache (0.018), seizures (0.017), lightheadedness (0.017), stiffness (0.013), double vision (0.013), hand numbness (0.008), eye pain (0.007), mental confusion (0.007), vision loss (0.006), throbbing headache (0.006), ache (0.006), crying (0.006), sharp pain (0.006), slurred speech (0.005), confusion (0.005), diarrhea (0.005), facial pain (0.005), sensitivity to sound (0.004), insomnia (0.004), severe pain (0.004), dehydration (0.004), partial loss of vision (0.004), stiff neck (0.004), difficulty speaking (0.003), persistent headache (0.003)
miscarriage	bleeding (0.471), vaginal bleeding (0.431), cramping (0.421), spotting (0.202), pain (0.149), abdominal pain (0.092), lower abdominal pain (0.038), nausea (0.037), dizziness (0.030), pelvic pain (0.027), lightheadedness (0.026), vaginal discharge (0.024), pain in lower abdomen (0.018), discomfort (0.015), pain during urination (0.014), cyst (0.013), irregular menstruation (0.009), cavity (0.007), blood in urine (0.007), severe pain (0.007), abdominal discomfort (0.007), sharp pain (0.006)
mono	sore throat (0.391), fever (0.280), pain (0.242), chills (0.192), lethargy (0.164), fatigue (0.155), coughing (0.111), difficulty swallowing (0.099), swelling (0.099), nausea (0.098), malaise (0.081), leaked fluid out of blood vessels or an organ (0.067), headache (0.066), painful swallowing (0.053), diarrhea (0.045), neck pain (0.045), redness (0.044), ear pain (0.042), enlarged neck lymph nodes (0.035), skin rash (0.035), fast heart rate (0.034), swollen lymph nodes (0.033), vomiting (0.033), discomfort (0.032), dry cough (0.029), infection (0.025), neck swelling (0.022), weakness (0.019), loss of appetite (0.018), stiffness (0.017), dizziness (0.017), tenderness (0.017)

multiple myeloma	back pain (0.070), fever (0.064), pain (0.055), weakness (0.052), lesion (0.046), coughing (0.040), diarrhea (0.035), chills (0.031), shortness of breath (0.025), abdominal pain (0.024), mental confusion (0.022), lethargy (0.021), cough with phlegm (0.020), hip pain (0.020), confusion (0.020), low blood pressure (0.019), leg pain (0.016), constipation (0.013)
multiple sclerosis	weakness (0.152), acute episodes (0.120), numbness (0.063), lethargy (0.039), seizures (0.021), fatigue (0.020), headache (0.020), leg pain (0.018), falling (0.018), incontinence (0.016), back pain (0.015), confusion (0.014), slurred speech (0.011), arm pain (0.011), double vision (0.011), difficulty walking (0.010), infection (0.010), blurred vision (0.010), neck pain (0.010), leaking of urine (0.010), leg numbness (0.010), mental confusion (0.009), lesion (0.008), hip pain (0.008), fast heart rate (0.008), diarrhea (0.007), constipation (0.007), muscle spasms (0.007), urinary retention (0.006), burning sensation (0.006), crying (0.006), hand numbness (0.005), weight loss (0.005), fever (0.005), difficulty swallowing (0.005), tremor (0.005), bruising (0.004), sensitivity to light (0.004), dehydration (0.004), problems with coordination (0.004), tenderness (0.004), hand pain (0.004), stiffness (0.004), insomnia (0.004), discomfort (0.004), difficulty raising the foot (0.004), nasal congestion (0.004), rapid involuntary eye movement (0.004), sleepiness (0.004), abnormality walking (0.004), vision loss (0.003), unsteady gait (0.003)
myasthenia gravis	weakness (0.158), difficulty swallowing (0.104), acute episodes (0.094), shortness of breath (0.083), double vision (0.077), drooping of upper eyelid (0.071), lethargy (0.067), coughing (0.056), fatigue (0.040), infection (0.029), fainting (0.022), low oxygen in the body (0.021), difficulty walking (0.021), slurred speech (0.020), incontinence (0.020), constipation (0.019), muscle weakness (0.018)
neutropenia	fever (0.344), chills (0.113), lethargy (0.085), fatigue (0.078), diarrhea (0.069), weakness (0.066), coughing (0.064), nausea (0.060), infection (0.050), vomiting (0.036), abdominal pain (0.031), malaise (0.031)
obesity	pain (0.167), nausea (0.060), sadness (0.042), depression (0.042), abdominal pain (0.035), tenderness (0.026), chills (0.026), diarrhea (0.024), shortness of breath (0.023), anxiety (0.023), back pain (0.020), vomiting (0.017), infection (0.016), leg pain (0.015), knee pain (0.014), swelling (0.012), chronic back pain (0.011), weakness (0.010), redness (0.009)
osteoarthritis	pain (0.175), knee pain (0.074), sadness (0.073), depression (0.073), hip pain (0.050), swelling (0.047), back pain (0.041), leg pain (0.038), bone loss (0.028), leaking of urine (0.024), anxiety (0.022), chronic back pain (0.021), shoulder pain (0.019), constipation (0.019), incontinence (0.015), tenderness (0.012), restless legs syndrome (0.011), stiffness (0.011)
osteoporosis	pain (0.186), back pain (0.088), depression (0.040), sadness (0.040), hip pain (0.039), weakness (0.034), lesion (0.028), anxiety (0.020), vertigo (0.017), constipation (0.016), numbness (0.016), leg pain (0.015)
ovarian cancer	pain (0.286), nausea (0.178), abdominal pain (0.147), vomiting (0.106), fever (0.054), weakness (0.053), fluid in the abdomen (0.051), diarrhea (0.050), constipation (0.044), chills (0.039), discomfort (0.031), shortness of breath (0.029), bowel obstruction (0.028), lethargy (0.024), fatigue (0.021), cramping (0.020), tenderness (0.019), dehydration (0.018), skin rash (0.015), crying (0.015), infection (0.013)
ovarian cyst	pain (0.785), nausea (0.303), cyst (0.198), abdominal pain (0.192), tenderness (0.115), vomiting (0.089), pelvic pain (0.087), diarrhea (0.069), lower abdominal pain (0.067), chills (0.058), vaginal bleeding (0.049), cramping (0.043), bleeding (0.039), vaginal discharge (0.037), spotting (0.036), pain during urination (0.029), discomfort (0.025), pain in lower abdomen (0.025), sharp pain (0.022), flank pain (0.021), constipation (0.018), fever (0.016), anxiety (0.013), pain in right lower abdomen (0.011), crying (0.010)
pancreatitis	pain (0.709), nausea (0.328), abdominal pain (0.326), vomiting (0.196), pain in upper abdomen (0.158), diarrhea (0.095), acute episodes (0.069), chills (0.055), tenderness (0.028), cyst (0.016), upper abdominal pain (0.015), vomiting blood (0.012), weight loss (0.009), depression (0.009), loss of appetite (0.009), sadness (0.009), fever (0.008), constipation (0.008), flank pain (0.008), severe pain (0.008), yellow skin and eyes (0.007), abdominal tenderness (0.006), abdominal discomfort (0.006)

parkinson's disease	weakness (0.075), confusion (0.049), tremor (0.039), mental confusion (0.035), lethargy (0.027), constipation (0.022), low blood pressure (0.017), agitation (0.017), hip pain (0.016), unsteady gait (0.012), delirium (0.011), slurred speech (0.011), falling (0.010), difficulty swallowing (0.008), fainting (0.008), visual hallucinations (0.008), tremor at rest (0.008), infection (0.008), coughing (0.007), dizziness (0.006), shortness of breath (0.006), difficulty walking (0.006), leaking of urine (0.006), urinary retention (0.006), malaise (0.005), cough with phlegm (0.005), incontinence (0.005), confusion in the evening hours (0.004), fatigue (0.004), hand tremor (0.004), failure to thrive (0.003), rectal pain (0.003), weight loss (0.003), tenderness (0.003), stiffness (0.003), difficulty speaking (0.003), difficulty breathing (0.003), restless legs syndrome (0.003), chronic cough (0.003)
pink eye	redness (0.318), eye redness (0.271), eye pain (0.163), itching (0.135), swelling (0.133), coughing (0.104), sore throat (0.088), discomfort (0.082), eye irritation (0.069), nasal congestion (0.048), fever (0.041), sensitivity to light (0.039), blurred vision (0.038), leaked fluid out of blood vessels or an organ (0.035), skin rash (0.028), cough with phlegm (0.027), headache (0.025), chills (0.024), eye discharge (0.021), infection (0.017), stuffy nose (0.011), swelling of the surface of the eye (0.010), dryness (0.010), facial swelling (0.010), watery eyes (0.010)
plantar fasciitis	heel pain (0.712), pain (0.485), foot pain (0.233), swelling (0.092), ankle pain (0.083), tenderness (0.058), limping (0.017), leg pain (0.017), bruising (0.013), sharp pain (0.010), severe pain (0.009), blood in urine (0.009), difficulty raising the foot (0.008), redness (0.008), mild pain (0.007), scar (0.007), blister (0.007)
pleural effusion	shortness of breath (0.186), coughing (0.116), low oxygen in the body (0.047), lethargy (0.036), fluid in the abdomen (0.032), cough with phlegm (0.032), infection (0.025), weakness (0.024), fluid in the lungs (0.023), fever (0.023), dry cough (0.022), fatigue (0.022), wheezing (0.021), collapse (0.020)
pneumonia	coughing (0.402), fever (0.208), shortness of breath (0.156), cough with phlegm (0.138), chills (0.091), lethargy (0.063), low oxygen in the body (0.060), weakness (0.043), wheezing (0.042), fatigue (0.037), infection (0.034), dry cough (0.030), malaise (0.030), fast heart rate (0.027), confusion (0.024), phlegm (0.018), respiratory distress (0.018), low blood pressure (0.017), fast breathing (0.016), chest pain worsened by breathing (0.014), mental confusion (0.014), coughing up blood (0.013), diarrhea (0.012), sore throat (0.010), difficulty breathing (0.010), night sweats (0.009), persistent cough (0.009), fluid in the lungs (0.008)
pneumothorax	shortness of breath (0.116), pain (0.086), rib pain (0.053), coughing (0.050), chest pain worsened by breathing (0.033), discomfort (0.022), bruising (0.018), collapse (0.017), back pain (0.014), seizures (0.013), fast heart rate (0.012), chest discomfort (0.011), cavity (0.010), infection (0.009), wheezing (0.009), difficulty breathing (0.009), shoulder pain (0.009), confusion (0.008), respiratory distress (0.008), chronic cough (0.008)
polycystic ovary syndrome	pain (0.412), nausea (0.177), cramping (0.080), abdominal pain (0.072), diarrhea (0.069), vaginal bleeding (0.061), pelvic pain (0.060), depression (0.059), sadness (0.058), dizziness (0.054), chills (0.051), bleeding (0.041), vomiting (0.040), anxiety (0.039), cyst (0.039), headache (0.038), lightheadedness (0.038), tenderness (0.033), irregular menstruation (0.030), sharp pain (0.030)
post traumatic stress disorder	sadness (0.380), depression (0.379), anxiety (0.284), thoughts of suicide (0.066), seizures (0.057), crying (0.040), insomnia (0.025), agitation (0.022), substance abuse (0.018), visual hallucinations (0.016), hearing voices (0.016), chronic back pain (0.015), back pain (0.015), paranoia (0.015), diarrhea (0.015), abdominal pain (0.014), screaming (0.013), nightmares (0.013), anger (0.013), panic attack (0.012), loss of appetite (0.010), falling (0.010), racing thoughts (0.010), tremor (0.009), confusion (0.007), itching (0.006), fear (0.006), bruising (0.005), hopelessness (0.005), fainting (0.004), severe anxiety (0.004), delirium (0.004), vision loss (0.003), poor appetite (0.003)
prostate cancer	weakness (0.077), blood in urine (0.062), urinary retention (0.043), pain (0.036), bleeding (0.036), constipation (0.032), infection (0.024), chills (0.023), lethargy (0.023), fever (0.023), incontinence (0.022), discomfort (0.022), back pain (0.017), bleeding from anus (0.016), fatigue (0.016), low blood pressure (0.015), leaking of urine (0.014), abdominal pain (0.014)
psoriasis	sadness (0.066), depression (0.062), skin rash (0.056), redness (0.056), anxiety (0.055), swelling (0.047), chills (0.038), diarrhea (0.032), infection (0.028), lesion (0.022), itching (0.021), pain (0.019), nausea (0.018), fever (0.018), abdominal pain (0.016), lump (0.016), poor appetite (0.015), dry cough (0.014), pain during urination (0.013), plaque (0.012), joint pain (0.011), lightheadedness

	(0.011), red rashes (0.011), dry skin (0.011)
psychosis	depression (0.206), sadness (0.206), agitation (0.105), anxiety (0.087), paranoia (0.083), hearing voices (0.060), visual hallucinations (0.039), thoughts of suicide (0.035), confusion (0.032), screaming (0.032), crying (0.023), insomnia (0.021), mental confusion (0.019), wandering and getting lost (0.017), substance abuse (0.017), delirium (0.017), racing thoughts (0.016), manic episode (0.014), seizures (0.014), aggression (0.012), fast heart rate (0.010), dehydration (0.008), delusion (0.008), hallucination (0.007), fear (0.007), falling (0.006), disorganized behavior (0.004), anger (0.004), tremor (0.004), nightmares (0.004), difficulty with bodily movement (0.004), weight loss (0.004), slurred speech (0.004)
pulmonary embolism	shortness of breath (0.244), fast heart rate (0.056), chest pain worsened by breathing (0.049), racing heartbeat (0.037), swelling (0.036), coughing (0.036), low oxygen in the body (0.030), fast breathing (0.026), discomfort (0.023), calf pain (0.023), infection (0.020), chest discomfort (0.019), leg pain (0.019), back pain (0.018), chest tightness (0.018), chills (0.018), lump (0.017), coughing up blood (0.015)
pulmonary hypertension	shortness of breath (0.160), regurgitation (0.150), low oxygen in the body (0.053), coughing (0.039), fluid in the lungs (0.029), back pain (0.028), restless legs syndrome (0.021), infection (0.021), wheezing (0.018), respiratory distress (0.015), chest discomfort (0.014), cavity (0.014), malnutrition (0.013), slow heart rate (0.012), dry cough (0.012), drooping of upper eyelid (0.012), vertigo (0.011), cough with phlegm (0.011), depression (0.010), sadness (0.010)
retinal detachment	vision loss (0.125), blurred vision (0.065), headache (0.057), neck pain (0.041), eye pain (0.039), dehydration (0.024), difficulty walking (0.023), itching (0.020), discomfort (0.018), unequal pupils (0.017), watery diarrhea (0.015), bone loss (0.015), partial loss of vision (0.014), ear pain (0.013), fast heart rate (0.012), slow bodily movement (0.009), low oxygen in the body (0.009), vision disorder (0.009), elevated alkaline phosphatase (0.009), seeing spots (0.009), abnormality walking (0.009), malnutrition (0.009)
rhabdomyolysis	dark urine (0.060), weakness (0.056), arm pain (0.055), muscle pain (0.045), swelling (0.043), cramping (0.030), leg pain (0.029), falling (0.024), numbness (0.022), bruising (0.022), lethargy (0.021), dehydration (0.020), substance abuse (0.018), confusion (0.017), sweating (0.016), kidney failure (0.016), fatigue (0.015), muscle weakness (0.015), blood in urine (0.015), seizures (0.015), slurred speech (0.015)
rheumatoid arthritis	pain (0.211), swelling (0.072), fever (0.043), knee pain (0.038), joint pain (0.033), infection (0.029), acute episodes (0.026), weakness (0.022), shortness of breath (0.021), diarrhea (0.020), shoulder pain (0.018), redness (0.017), fatigue (0.016), hip pain (0.015), chills (0.015), depression (0.013), sadness (0.013), lethargy (0.013), wrist pain (0.013), fast heart rate (0.012), skin rash (0.011), low blood pressure (0.010)
schizophrenia	hearing voices (0.091), depression (0.069), sadness (0.069), paranoia (0.055), agitation (0.047), anxiety (0.033), thoughts of suicide (0.026), visual hallucinations (0.021), wandering and getting lost (0.015), screaming (0.014), confusion (0.014), aggression (0.011), seizures (0.011), substance abuse (0.010), racing thoughts (0.009), difficulty with bodily movement (0.008), crying (0.008), insomnia (0.008), tremor (0.007), low blood pressure (0.006), fast heart rate (0.006), slurred speech (0.005), panic attack (0.005), anger (0.005), low oxygen in the body (0.004), mental confusion (0.004), drooling (0.004), excess thirst (0.003), fear (0.003), wheezing (0.003)
sciatica	pain (0.627), back pain (0.344), leg pain (0.144), numbness (0.106), hip pain (0.076), chronic back pain (0.050), weakness (0.047), incontinence (0.023), tenderness (0.018), difficulty walking (0.016), leaking of urine (0.015), limping (0.015), leg numbness (0.013), constipation (0.011), severe pain (0.011), lower extremity pain (0.011), foot numbness (0.011), crying (0.010), acute episodes (0.009), sharp pain (0.009), flank pain (0.009), pins and needles (0.009), knee pain (0.008), difficulty raising the foot (0.007), groin pain (0.007), nerve pain (0.006)

seasonal allergies	coughing (0.122), nasal congestion (0.077), sore throat (0.070), headache (0.054), swelling (0.043), shortness of breath (0.042), cough with phlegm (0.034), redness (0.032), stuffy nose (0.032), runny nose (0.031), itching (0.030), wheezing (0.030), sneezing (0.027), anxiety (0.026), chills (0.022), dry cough (0.022), chest tightness (0.021), discomfort (0.021), sinus pressure (0.020), skin rash (0.019), eye redness (0.019), itchy eyes (0.018), ear pain (0.018), racing heartbeat (0.016), sadness (0.014), depression (0.014), fatigue (0.013), difficulty breathing (0.013), dizziness (0.013)
sebaceous cyst	cyst (0.316), swelling (0.181), pain (0.161), lump (0.151), redness (0.116), lesion (0.060), muscle twitches (0.045), brief visual or sensory abnormality (0.033), bone loss (0.030), discomfort (0.029), drooping of upper eyelid (0.025), fast heart rate (0.022)
sepsis	fever (0.272), low blood pressure (0.174), infection (0.074), chills (0.072), fast heart rate (0.068), confusion (0.063), lethargy (0.051), weakness (0.047), coughing (0.039), low oxygen in the body (0.027), mental confusion (0.024), kidney failure (0.022), diarrhea (0.020), respiratory distress (0.014), malaise (0.014), dehydration (0.013), redness (0.013), fast breathing (0.011), abdominal pain (0.011), shortness of breath (0.010), vomiting (0.009), fatigue (0.008), low body temperature (0.006), wheezing (0.006), collapse (0.005), malnutrition (0.005), fluid in the abdomen (0.005), difficulty breathing (0.005)
shingles	skin rash (0.301), pain (0.278), nerve pain (0.047), itching (0.032), redness (0.031), flank pain (0.029), lesion (0.027), back pain (0.026), weakness (0.026), infection (0.024), lethargy (0.019), facial pain (0.016), sensitivity to light (0.016), ear pain (0.015), red rashes (0.015), eye pain (0.013), fatigue (0.012), arm pain (0.012), blister (0.011), rash on the face (0.008), facial swelling (0.008), swelling (0.008), stiffness (0.008), sharp pain (0.007), blurred vision (0.007), burning sensation (0.007), inflammation of ear (0.007), neck pain (0.005), weight loss (0.005)
sickle cell anemia	pain (0.689), back pain (0.147), leg pain (0.100), arm pain (0.068), hip pain (0.056), knee pain (0.050), fever (0.044), joint pain (0.043), seizures (0.038), infection (0.033), shortness of breath (0.030), malnutrition (0.028), chest pain worsened by breathing (0.025), acute episodes (0.024), discomfort (0.022), severe pain (0.022), rib pain (0.021), sharp pain (0.020), coughing up blood (0.017), coughing (0.015), itching (0.015), vomiting (0.015), lower extremity pain (0.014), nausea (0.013), elbow pain (0.013), pelvic pain (0.012), vomiting blood (0.012)
sinusitis	headache (0.172), coughing (0.144), fever (0.097), chills (0.082), nausea (0.079), nasal congestion (0.068), sinus pressure (0.062), sinus pain (0.061), dizziness (0.051), pain (0.044), sore throat (0.039), facial pain (0.037), tenderness (0.036), ear pain (0.029), infection (0.028), sensitivity to light (0.027), cough with phlegm (0.026), diarrhea (0.025), lethargy (0.024), fatigue (0.021), skin rash (0.019), neck pain (0.018), eye pain (0.018), stuffy nose (0.017), lightheadedness (0.015), inflammation of ear (0.014), blurred vision (0.014), discomfort (0.014), malaise (0.013), dry cough (0.013), vertigo (0.012), jaw pain (0.011), phlegm (0.011), shortness of breath (0.010), double vision (0.010)
sleep apnea	depression (0.086), sadness (0.086), shortness of breath (0.045), lethargy (0.044), anxiety (0.032), fatigue (0.028), coughing (0.024), snoring (0.014), confusion (0.014), chronic cough (0.013), falling (0.013), mental confusion (0.011), chills (0.010), erectile dysfunction (0.010), malaise (0.009), abdominal pain (0.009), bone loss (0.009), dizziness (0.009), infection (0.008), cough with phlegm (0.008), episodes of no breathing (0.008), skin rash (0.008), seizures (0.008), severe pain (0.008), regurgitation (0.007)
spondylosis	pain (0.254), weakness (0.115), back pain (0.104), neck pain (0.092), numbness (0.091), headache (0.064), sadness (0.063), depression (0.063), dizziness (0.043), incontinence (0.041), fainting (0.038), shoulder pain (0.037), lesion (0.033), chronic back pain (0.032), drooping of upper eyelid (0.032), tenderness (0.025), arm pain (0.023), anxiety (0.023)
sprained ankle	pain (0.504), swelling (0.461), ankle pain (0.413), tenderness (0.060), foot pain (0.052), bruising (0.037), limping (0.033), falling (0.027), physical deformity (0.014), difficulty walking (0.014), ice pack (0.013), calf pain (0.008), throbbing pain (0.007), severe pain (0.006), discomfort (0.006)
sprained knee	knee pain (0.495), pain (0.425), swelling (0.193), calf pain (0.049), leg pain (0.037), limping (0.035), tenderness (0.026), discomfort (0.020), falling (0.017), bruising (0.016), severe pain (0.013), ankle pain (0.012), ice pack (0.011), shoulder pain (0.010), difficulty walking (0.010), sharp pain (0.010), swollen joint (0.007)
sprained wrist	pain (0.402), wrist pain (0.323), swelling (0.308), hand pain (0.103), tenderness (0.072), falling (0.060), knee pain (0.033), physical deformity (0.029), finger pain (0.029), bruising (0.026), arm

	pain (0.018), numbness (0.016), elbow pain (0.015), ice pack (0.014), mild pain (0.009), ankle pain (0.009), decreased range of motion (0.009)
stomach flu	diarrhea (0.533), nausea (0.526), vomiting (0.474), pain (0.336), abdominal pain (0.242), chills (0.166), cramping (0.112), fever (0.089), dehydration (0.066), watery diarrhea (0.063), tenderness (0.053), pain in upper abdomen (0.041), dizziness (0.041), abdominal discomfort (0.028), discomfort (0.028), lethargy (0.027), weakness (0.026), fatigue (0.025), headache (0.023), lightheadedness (0.019), lower abdominal pain (0.015), malaise (0.011), low blood pressure (0.010), pain during urination (0.010), fast heart rate (0.009), blood in stool (0.008), abdominal tenderness (0.008), upper abdominal pain (0.008), anxiety (0.008), loss of appetite (0.008), muscle pain (0.006), dark stool from digested blood (0.006), intermittent abdominal pain (0.006), acute episodes (0.006), gagging (0.006), constipation (0.006), stomach cramps (0.005), discomfort in upper abdomen (0.005)
stomach ulcer	pain (0.254), abdominal pain (0.120), nausea (0.119), sadness (0.109), depression (0.109), pain in upper abdomen (0.083), vomiting (0.074), diarrhea (0.037), bleeding (0.036), tenderness (0.034), vomiting blood (0.031), chills (0.026), malnutrition (0.018), seizures (0.013), lightheadedness (0.012), abdominal discomfort (0.011), dizziness (0.011), indigestion (0.011), wrist pain (0.010), shortness of breath (0.009), bowel obstruction (0.009)
strep throat	sore throat (0.605), fever (0.347), pain (0.225), chills (0.215), coughing (0.159), leaked fluid out of blood vessels or an organ (0.127), difficulty swallowing (0.080), headache (0.075), ear pain (0.062), redness (0.060), swelling (0.058), nausea (0.051), neck pain (0.048), infection (0.037), nasal congestion (0.036), fatigue (0.036), painful swallowing (0.035), lethargy (0.033), vomiting (0.027), dry cough (0.026), tenderness (0.023), diarrhea (0.021), runny nose (0.021), skin rash (0.021), swollen tonsils (0.018), malaise (0.017), irritation of the tonsils (0.017), neck swelling (0.017), stiffness (0.016), night sweats (0.015), difficulty breathing (0.015)
stroke	weakness (0.293), slurred speech (0.098), numbness (0.089), dizziness (0.078), headache (0.076), confusion (0.076), mental confusion (0.055), seizures (0.044), vertigo (0.028), malnutrition (0.027), weakness of one side of the body (0.026), blurred vision (0.022), problems with coordination (0.021), unsteady gait (0.021), difficulty speaking (0.015), double vision (0.013), vision loss (0.013), hand numbness (0.012), falling (0.012), fainting (0.011), lightheadedness (0.011), paralysis (0.010), difficulty swallowing (0.010), lethargy (0.009), unsteadiness (0.008), rapid involuntary eye movement (0.008), memory loss (0.007), clumsiness (0.007), lesion (0.007), drooling (0.006)
tension headache	headache (0.613), pain (0.227), nausea (0.149), neck pain (0.123), dizziness (0.119), sensitivity to light (0.071), lightheadedness (0.057), anxiety (0.042), stiffness (0.040), chills (0.037), ache (0.036), blurred vision (0.036), vertigo (0.032), racing heartbeat (0.030), weakness (0.024), rapid involuntary eye movement (0.022), fainting (0.020), poor appetite (0.018), nasal congestion (0.017), vision loss (0.017), foot pain (0.016), insomnia (0.016)
thoracic radiculopathy	pain (0.621), back pain (0.477), numbness (0.335), weakness (0.229), leg pain (0.169), hip pain (0.098), chronic back pain (0.098), incontinence (0.061), foot numbness (0.054), leg numbness (0.053), pins and needles (0.038), severe pain (0.034), sharp pain (0.025), tenderness (0.024), leaking of urine (0.023), difficulty walking (0.023), difficulty raising the foot (0.022), knee pain (0.018), flank pain (0.016)
thyroid cancer	nausea (0.097), weakness (0.067), headache (0.064), coughing (0.050), fever (0.044), diarrhea (0.042), chills (0.042), dizziness (0.039), lethargy (0.037), fatigue (0.035), numbness (0.030), vomiting (0.030), racing heartbeat (0.026), difficulty swallowing (0.023)
thyroid nodules	pain (0.085), back pain (0.065), discomfort (0.055), fainting (0.051), bone loss (0.040), dizziness (0.039), difficulty swallowing (0.036), lump (0.036), swelling (0.031), racing heartbeat (0.031), headache (0.031), neck pain (0.027)
traumatic brain injury	headache (0.135), dizziness (0.060), seizures (0.059), fainting (0.051), nausea (0.048), tenderness (0.042), bruising (0.041), falling (0.039), neck pain (0.038), confusion (0.032), bleeding (0.029), mental confusion (0.027), blurred vision (0.026), vomiting (0.016), agitation (0.014), slurred speech (0.012), vertigo (0.011), double vision (0.010), memory loss (0.010), sensitivity to light (0.010), unsteady gait (0.008), amnesia (0.008), persistent headache (0.008), problems with coordination (0.007), severe headache (0.007), difficulty concentrating (0.006), lightheadedness (0.006), rapid involuntary eye movement (0.006), elbow pain (0.006), stiffness (0.004), eye pain (0.004), jaw pain (0.004), rib pain (0.004), malnutrition (0.003), sleepiness (0.003), paralysis (0.003), shoulder pain

	(0.003), brief visual or sensory abnormality (0.003), substance abuse (0.003)
type 1 diabetes	nausea (0.043), vomiting (0.039), infection (0.023), chills (0.022), lethargy (0.019), weakness (0.018), diarrhea (0.015), excess urination (0.012), fatigue (0.012), fever (0.009), dizziness (0.009), excess thirst (0.008), pain during urination (0.008), increased thirst (0.007), shortness of breath (0.007), foot pain (0.007), abdominal pain (0.006), malaise (0.006), kidney failure (0.006), coughing (0.006), thirst (0.006), depression (0.006)
type 2 diabetes	weakness (0.029), infection (0.014), lethargy (0.013), dizziness (0.012), fatigue (0.009), redness (0.008), excess urination (0.007), confusion (0.006), leg pain (0.005), foot pain (0.005), shortness of breath (0.005), mental confusion (0.005), low blood pressure (0.004), excess thirst (0.004), coughing (0.003), increased thirst (0.003), malaise (0.003)
ulcerative colitis	pain (0.414), diarrhea (0.278), abdominal pain (0.248), nausea (0.228), acute episodes (0.215), vomiting (0.097), cramping (0.084), fever (0.082), chills (0.066), blood in stool (0.038), bleeding from anus (0.035), tenderness (0.033), lower abdominal pain (0.028), scarring within the bile ducts (0.027), weight loss (0.024), fatigue (0.023), lethargy (0.019), bowel obstruction (0.019), rectal pain (0.019), dehydration (0.019), infection (0.018), bleeding (0.018), fast heart rate (0.018), lightheadedness (0.016), discomfort (0.013), constipation (0.013)
upper respiratory infection	coughing (0.503), fever (0.187), sore throat (0.165), cough with phlegm (0.163), shortness of breath (0.148), chills (0.124), runny nose (0.094), wheezing (0.080), nasal congestion (0.074), headache (0.061), ear pain (0.046), dry cough (0.041), vertigo (0.038), redness (0.037), leaking of urine (0.032), mild cough (0.028), malaise (0.027), sneezing (0.027), pain during urination (0.026), loss of appetite (0.026), lethargy (0.025), fatigue (0.024), chest pressure (0.024), acute episodes (0.024), tenderness (0.024)
urinary tract infection	blood in urine (0.146), pain during urination (0.146), fever (0.071), chills (0.045), flank pain (0.043), urinary retention (0.043), confusion (0.034), lethargy (0.028), weakness (0.028), pain in lower abdomen (0.022), mental confusion (0.021), infection (0.021), discomfort (0.020), nausea (0.015), abdominal pain (0.013), constipation (0.011), diarrhea (0.011), cloudy urine (0.010), frequent urge to urinate (0.010), fatigue (0.010), low blood pressure (0.010), incontinence (0.010), leaking of urine (0.009), malaise (0.009), foul smelling urine (0.008), back pain (0.008), lower abdominal pain (0.008), pelvic pain (0.008), tenderness (0.007), dehydration (0.006), dark urine (0.005), abdominal discomfort (0.005), vaginal discharge (0.004), fast heart rate (0.004), frequent urination (0.004), groin pain (0.004)
vitamin d deficiency	depression (0.119), sadness (0.118), malnutrition (0.096), anxiety (0.075), bone loss (0.056), seizures (0.055), diarrhea (0.046), vomiting (0.042), weight loss (0.031), dizziness (0.028), dehydration (0.027), infection (0.023), insomnia (0.021), fast heart rate (0.020), leaking of urine (0.019)

Appendix 3: Clinical Evaluation for Alternate Settings

In the clinical evaluation presented in the main paper we took physicians' tagging of potential edges on a 4-point scale ('always happens', 'sometimes happens', 'rarely happens' or 'never happens') and binarized them, grouping results from the 'always', 'sometimes' and 'rarely' categories into the positive category and leaving the 'never' tag to be negative. Below we present the precision-recall curve of an alternate binarization according to which results from the 'always' and 'sometimes' categories are grouped into the positive category and 'rarely' and 'never' tags are grouped into the negative. The figure below shows that the relative performance of the models is consistent across binarization schemas, with noisy or outperforming naïve Bayes on both precision and recall.

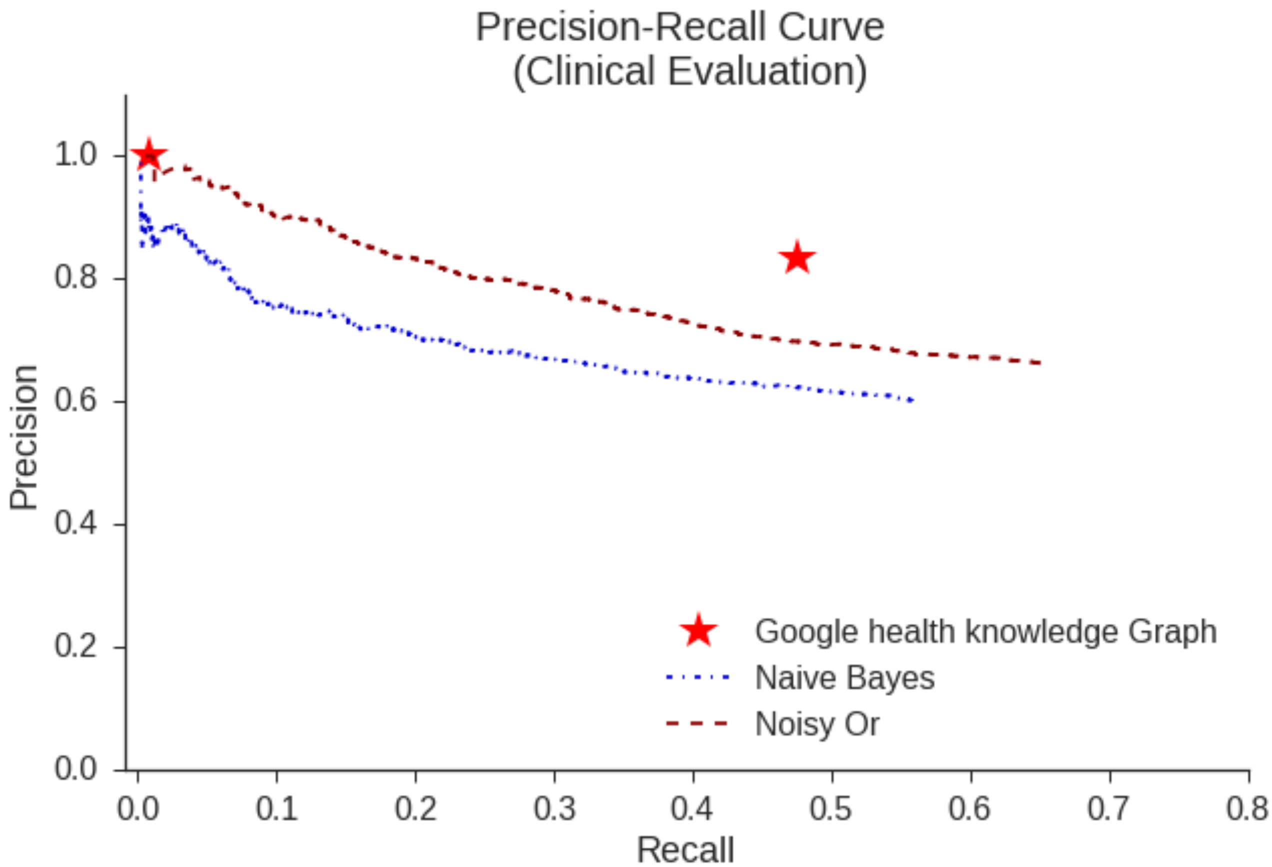

Figure 1: Precision-recall curves for the clinical evaluation framework with alternate binarization.

References

1. Anand, V. & Downs, S.M. Probabilistic asthma case finding: a noisy or reformulation. in *Amia* (2008).
2. Pearl, J. *Causality models, reasoning, and inference*, (Cambridge University Press,, Cambridge England ; New York, 2009).
